# Pathogen Adaptation of HLA Alleles and Its Correlation with Autoimmune Diseases in the Han Chinese

**DOI:** 10.1093/gpbjnl/qzaf038

**Published:** 2025-04-29

**Authors:** Shuai Liu, Yanyan Li, Tingrui Song, Jingjing Zhang, Peng Zhang, Huaxia Luo, Sijia Zhang, Yiwei Niu, Tao Xu, Shunmin He

**Affiliations:** State Key Laboratory of Epigenetic Regulation and Intervention, Institute of Biophysics, Chinese Academy of Sciences, Beijing 100101, China; University of Chinese Academy of Sciences, Beijing 100049, China; College of Life Sciences, University of Chinese Academy of Sciences, Beijing 100049, China; State Key Laboratory of Epigenetic Regulation and Intervention, Institute of Biophysics, Chinese Academy of Sciences, Beijing 100101, China; State Key Laboratory of Epigenetic Regulation and Intervention, Institute of Biophysics, Chinese Academy of Sciences, Beijing 100101, China; State Key Laboratory of Epigenetic Regulation and Intervention, Institute of Biophysics, Chinese Academy of Sciences, Beijing 100101, China; University of Chinese Academy of Sciences, Beijing 100049, China; State Key Laboratory of Epigenetic Regulation and Intervention, Institute of Biophysics, Chinese Academy of Sciences, Beijing 100101, China; State Key Laboratory of Epigenetic Regulation and Intervention, Institute of Biophysics, Chinese Academy of Sciences, Beijing 100101, China; State Key Laboratory of Epigenetic Regulation and Intervention, Institute of Biophysics, Chinese Academy of Sciences, Beijing 100101, China; University of Chinese Academy of Sciences, Beijing 100049, China; College of Life Sciences, University of Chinese Academy of Sciences, Beijing 100049, China; State Key Laboratory of Epigenetic Regulation and Intervention, Institute of Biophysics, Chinese Academy of Sciences, Beijing 100101, China; University of Chinese Academy of Sciences, Beijing 100049, China; College of Life Sciences, University of Chinese Academy of Sciences, Beijing 100049, China; University of Chinese Academy of Sciences, Beijing 100049, China; National Laboratory of Biomacromolecules, CAS Center for Excellence in Biomacromolecules, Institute of Biophysics, Chinese Academy of Sciences, Beijing 100101, China; Shandong First Medical University & Shandong Academy of Medical Sciences, Jinan 250117, China; State Key Laboratory of Epigenetic Regulation and Intervention, Institute of Biophysics, Chinese Academy of Sciences, Beijing 100101, China; University of Chinese Academy of Sciences, Beijing 100049, China; College of Life Sciences, University of Chinese Academy of Sciences, Beijing 100049, China

**Keywords:** HLA genotype, Han Chinese, HLA–peptide binding, Pathogen adaptation, Autoimmune disease

## Abstract

Human leukocyte antigen (HLA) genes play a crucial role in the adaptation of human populations to the dynamic pathogenic environment. Despite their significance, investigating the pathogen-driven evolution of HLAs and its implications for autoimmune diseases presents considerable challenges. Here, we genotyped over 20 HLA genes at 3-field resolution in 8278 individuals from diverse ethnic backgrounds, including 4013 unrelated Han Chinese individuals. We focused on the adaptation of HLAs in the Han Chinese population by analyzing their binding affinity for various pathogens, and explored the potential correlations between pathogen adaptation and autoimmune diseases. Our findings reveal that specific HLA alleles like HLA-DRB1*07:01 and HLA-DQB1*06:01 confer strong pathogen adaptability at the sequence level, notably for *Corynebacterium diphtheriae* and *Bordetella pertussis*. Additionally, alleles like HLA-C*03:02 demonstrate adaptive selection against pathogens like *Mycobacterium tuberculosis* and coronavirus at the gene expression level. Simultaneously, the aforementioned HLA alleles are closely related to some autoimmune diseases such as multiple sclerosis. These exploratory discoveries shed light on the intricate coevolutionary relationships between pathogen adaptation and autoimmune diseases in the human population. These efforts led to an HLA database at http://bigdata.ibp.ac.cn/HLAtyping, aiding searches for HLA allele frequencies across populations.

## Graphical abstract

**Graphical abstract:**
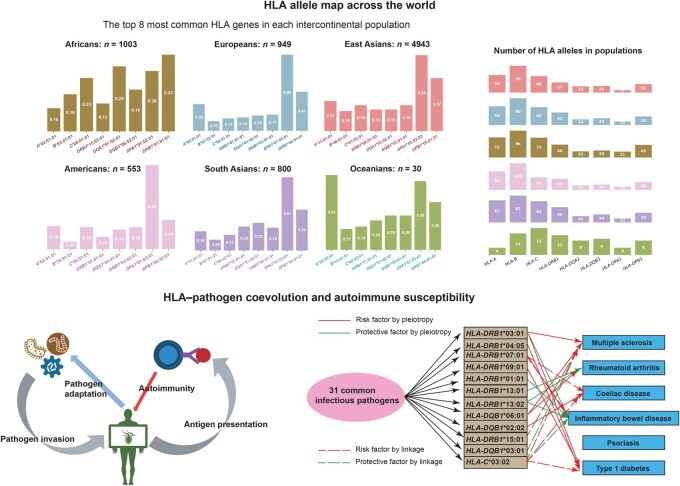

## Introduction

The immune process is of paramount importance in the intricate interplay between the *in vivo* life system and the surrounding pathogenic microbial environment [1]. The human leukocyte antigen (HLA) genes located on the chromosomal region 6p21.3, in particular the classical HLA genes (*HLA-A*, *HLA-B*, *HLA-C*, *HLA-DRB1*, *HLA-DPA1*, *HLA-DPB1*, *HLA-DQA1*, and *HLA-DQB1*), play a pivotal role in the recognition and presentation of invasive foreign pathogens to the immune system [2]. As a result, these classical HLA genes are under intense selective pressure for resistance against pathogens, resulting in the highest degree of polymorphism observed in the human genome [3,4]. To date, two primary mechanistic theories, negative frequency-dependent selection (NFDS) and heterozygote advantage (HA), have been proposed to explain the remarkable diversity of HLA genes in the fluctuating pathogenic microbial environment [5]. Genetic predisposition to different pathogens is an area of increasing interest [6]. Although many human HLA resources have been developed [7–14], the vast population of China necessitates expanding HLA genetic data resources with high-resolution genotyping for the Chinese population and focusing on human–pathogen coevolution. By analyzing the binding affinity of HLA molecules for pathogenic peptides, we can gain valuable insights into the evolutionary history of human adaptation to pathogens [15,16].

There has always been a phenomenon of coadaptation between humans and pathogens, which has been found to have an important impact on many human diseases, a typical example being autoimmune diseases. The hypothesis of “pathogen-driven selection” provides important insights into the mechanisms of the evolution of autoimmune diseases [17,18]. A study in ancient genomics reveals that immune-related genes have been strongly affected by both positive and negative selection, and that resistance to infection has increased the risk of inflammatory disease over the past millennia [19]. HLA genes play a crucial role in pathogen adaptation and autoimmunity. However, despite their importance, there is limited research utilizing HLA genes to explore how pathogen adaptation genetically impacts autoimmune diseases within populations.

In this study, we performed high-resolution genotyping of HLA alleles in a large cohort consisting of 4129 individuals from the NyuWa Genome Project [20], 3202 individuals from the 1000 Genomes Project (1KGP) [21], and 947 individuals from the Human Genome Diversity Project (HGDP) [22]. Our comprehensive analysis characterized the binding affinity of common HLA alleles in the Han Chinese population for a range of human epidemic pathogens, and evaluated the influence of pathogen adaptation of HLA alleles on autoimmune diseases. Finally, we explored the adaptive selection on the transcriptional regulation of the HLA alleles under the pathogen pressure. The analysis of HLA alleles in a large Han population helps us understand the adaptation landscape of populations to pathogens and the relationship between pathogen adaptation and autoimmune diseases.

## Results

### High-resolution genotyping of HLA genes in multiracial populations

**Figure 1:**
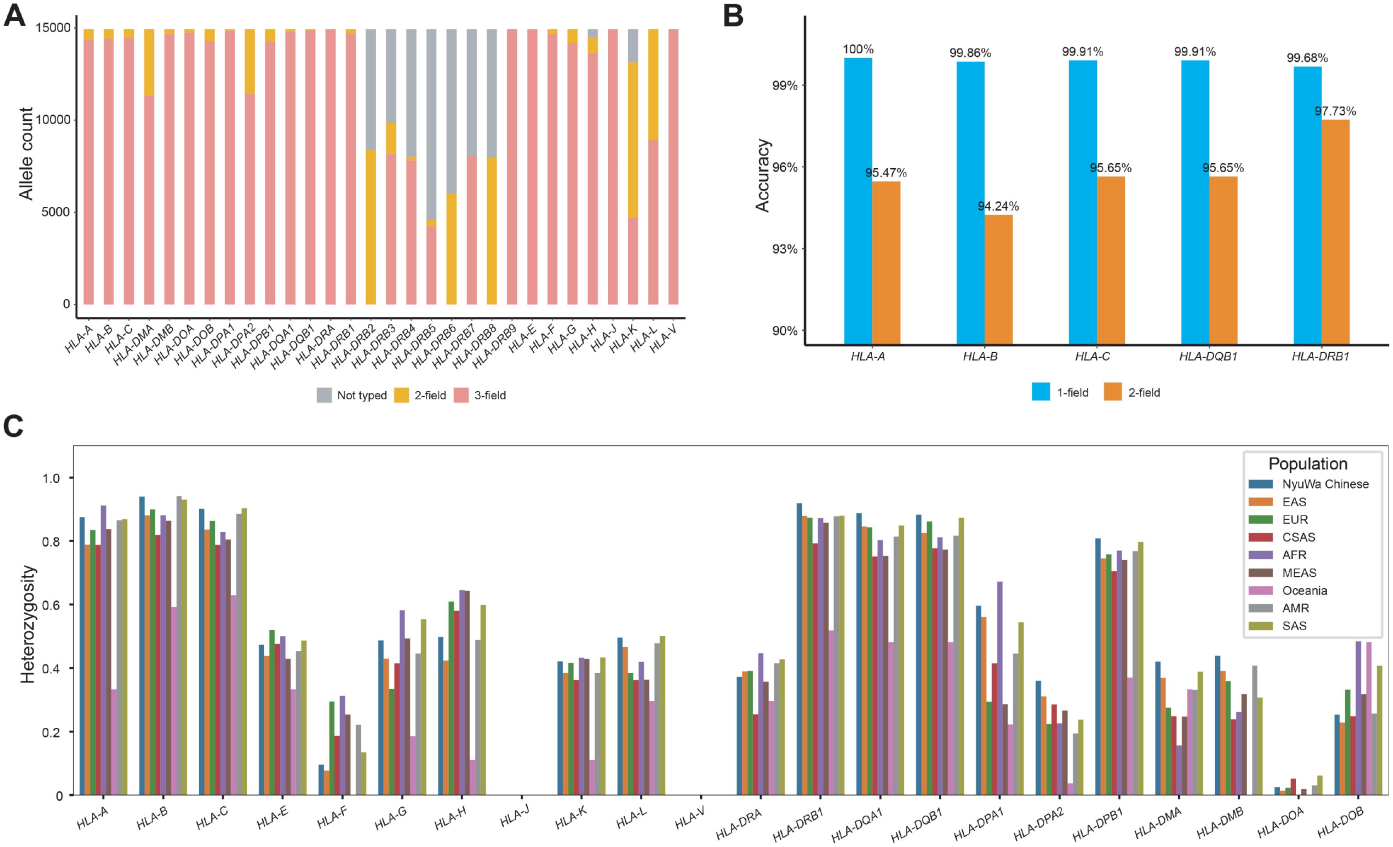
The evaluation and description for genotyped HLA genes **A**. Proportion of individuals with completed HLA genotyping and the resolution of genotyped HLA alleles. “Not typed” indicates cases where NGS reads could not be assigned to any known HLA allele or determined to be blank by HLA-HD software. **B**. Accuracy of HLA genotyping based on HLA-HD. This evaluation was performed based on the SBT-sequenced benchmark HLA genotypes from 1KGP. **C**. Heterozygosity of HLA genes in different populations. HLA, human leukocyte antigen; 1KGP, 1000 Genomes Project; NGS, next-generation sequencing; SBT, sequence-based typing; HLA-HD, HLA typing from High-quality Dictionary; EAS, East Asian; EUR, European; CSAS, Central South Asian; AFR, African; MEAS, Middle Eastern Asian; AMR, American; SAS, South Asian.

In this comprehensive study, we successfully genotyped a total of 31 HLA genes. Our analysis was grounded in the assessment of genotyping rate, resolution, and accuracy across 8278 samples from the NyuWa Genome Project, the 1KGP, and the HGDP. The genotyping rates of various HLA genes within these cohorts revealed that the classical HLA genes, including *HLA-A*, *HLA-B*, *HLA-C*, *HLA-DRB1*, *HLA-DQA1*, *HLA-DQB1*, *HLA-DPA1*, and *HLA-DPB1*, were genotyped in all samples (**Figure 1**A). Moreover, all these HLA genes achieved a minimum of 2-field genotyping level (amino acid level), with majority achieving the 3-field genotyping level (exon sequence level) (Figure 1A). Upon comparing our genotyping results with benchmark genotypes for *HLA-A*, *HLA-B*, *HLA-C*, *HLA-DRB1*, and *HLA-DQB1* from the 1KGP, we observed that the genotyping accuracy for these genes exceeded 99% at the 1-field genotyping level (serotyping level), and had an impressive 94%–97% accuracy at the amino acid level. (Figure 1B). In addition, we quantified the genetic diversity by calculating the heterozygosity of HLA genes with genotyping rates surpassing 80%. These findings indicate that the seven classical HLA genes, *HLA-A*, *HLA-B*, *HLA-C*, *HLA-DRB1*, *HLA-DQA1*, *HLA-DQB1*, and *HLA-DPB1*, exhibit exceptionally high heterozygosity, with values exceeding 80% within the population (Figure 1C). This high level of heterozygosity underscores their critical roles in the adaptation to pathogens [3,4]. The genetic architecture of HLA genes in the Han Chinese population closely resembles that of the East Asian population but exhibits significant differences when compared to the European and African populations. In particular, the most frequent HLA haplotype (HLA-A*02:07–HLA-C*01:02–HLA-B*46:01–HLA-DRA*01:01–HLA-DRB1*09:01–HLA-DQA1*03:02–HLA-DQB1*03:03–HLA-DPA1*02:02–HLA-DPB1*05:01) in the Han Chinese population only accounted for 0.19%, while the most frequent HLA haplotype (HLA-A*02:07–HLA-C*01:02–HLA-B*46:01–HLA-DRA*01:01–HLA-DRB1*14:54–HLA-DQA1*01:04–HLA-DQB1*05:02–HLA-DPA1*02:02–HLA-DPB1*02:02) in the East Asian population accounted for 0.30%, the most frequent HLA haplotype (HLA-A*03:01–HLA-C*07:02–HLA-B*07:02–HLA-DRA*01:02–HLA-DRB1*15:01–HLA-DQA1*01:02–HLA-DQB1*06:02–HLA-DPA1*01:03–HLA-DPB1*04:01) in the European population accounted for 0.40%, and the most frequent HLA haplotype (HLA-A*30:01–HLA-C*17:01–HLA-B*42:01–HLA-DRA*01:02–HLA-DRB1*03:02–HLA-DQA1*04:01–HLA-DQB1*04:02–HLA-DPA1*02:02–HLA-DPB1*01:01) in the African population accounted for 0.23% (Figure S1A–D).

The HLA allele resource in this study provided high-resolution HLA alleles for a large number of Han Chinese individuals, along with those from multi-ethnic populations worldwide. HLA alleles with 2-field or higher genotyping fields can provide information on the amino acid chain, which could help us complete molecular docking to determine the ability of HLA molecules to recognize foreign substances, and further find out the recent adaptation landscapes of HLA alleles in human populations. In addition, this HLA data resource, compared with the Allele Frequency Net Database [23], has supplemented many missing genotypes of HLA genes in the northern Han population, facilitating deeper insights into the diversity and adaptive evolution of HLA genes in the Han Chinese population.

### Pathogen–peptide binding landscape of HLA elements in the Han Chinese population

In order to ascertain potential correlations between HLA–peptide binding affinity and pathogen adaptation, an investigation was conducted to evaluate the impact of HLA–peptide binding affinity on the adaptability of the human immune system in the context of human immunodeficiency virus (HIV) and hepatitis C virus (HCV) infection. This investigation focused in particular on the associated HLA alleles as reported in previous studies [24,25]. The results indicate a significant correlation between an increased HLA binding affinity to HIV or HCV peptides and a reduced capacity for pathogen invasion and persistence (Figures S2 and S3). These findings reveal a critical aspect of HLA adaptation to these viruses, *i.e.*, a higher affinity confers an enhanced ability to identify and combat these pathogens. Although HIV and HCV have only been present in the human population for only a few decades, the population’s adaptation to the consensus sequences of other pathogens can lead to the adaptation of HIV and HCV [26,27]. On the other hand, to further demonstrate the correlations between HLA–peptide binding affinity and pathogen adaptation, we observed the correlation between the differences in the pathogen prevalence among different populations and the adaptive HLA frequencies. It can be seen that there are negative correlations between adaptive HLA frequencies and pathogen prevalence (Table S1). These discoveries have profound biological significance, as it underscores the strategic use of affinity scores of HLA molecules for pathogen-derived peptides in the prediction and analysis of immune responses, thereby enhancing our understanding of host–pathogen interaction and coevolution.

**Figure 2:**
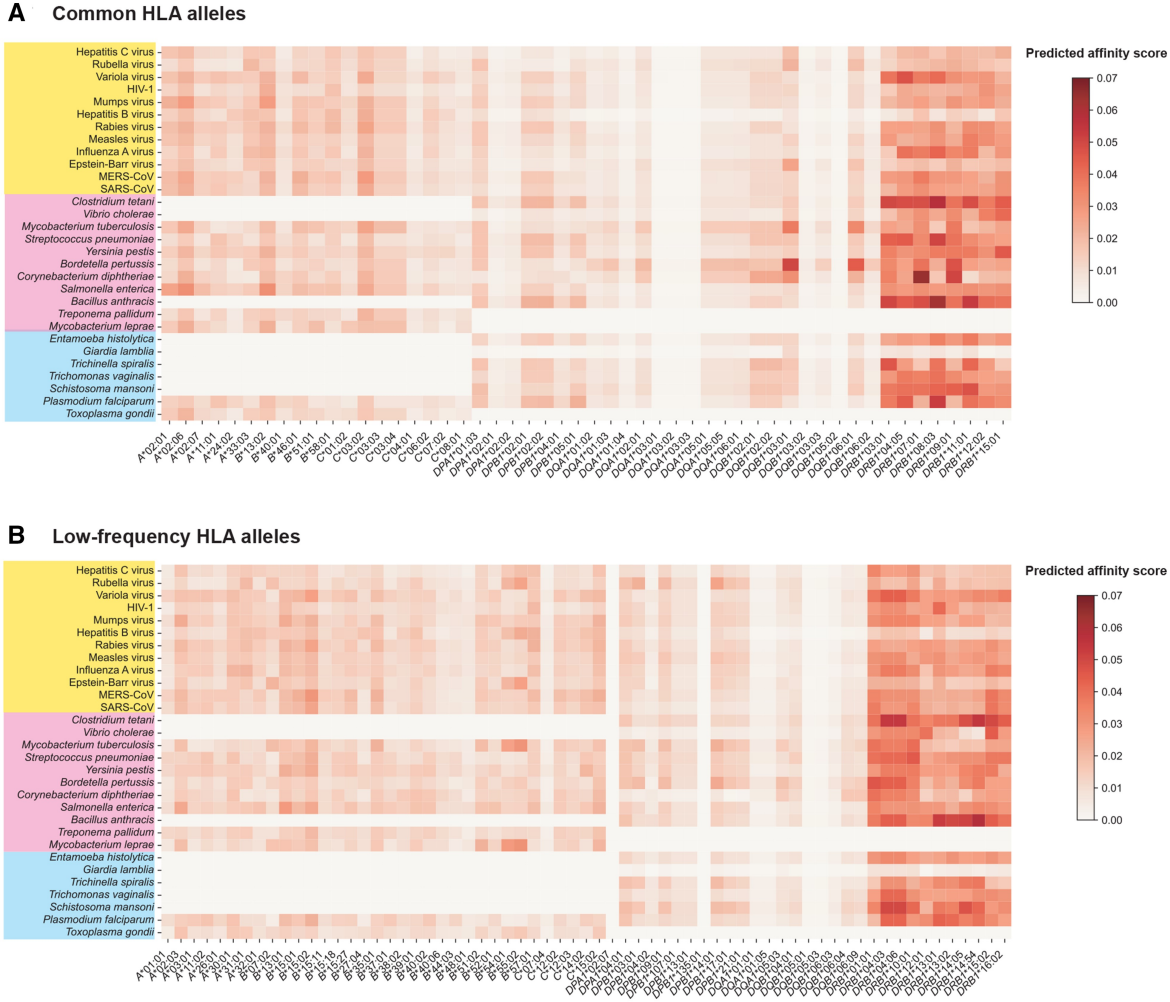
Binding affinity between HLA alleles and epidemic pathogens in the Han Chinese population **A**. Common HLA alleles. **B**. Low-frequency HLA alleles. Color coding indicates pathogen types: yellow (viruses), pink (bacteria), and blue (parasites). Higher scores indicate stronger affinity.

By evaluating the binding affinity of both the common HLA types [allele frequency (AF) ≥ 5%] and the low-frequency HLA types (AF ≥ 0.01 and AF < 0.05) with various epidemic pathogens (Table S2), we observed that the different alleles of *HLA-DRB1*, regardless of their AF, demonstrated a stronger ability to bind to a wide range of pathogen antigens (**Figure 2**A and B). This phenomenon is also observed for the rare *HLA-DRB1* alleles in the population (Figure S4), as well as for *HLA-DRB1* alleles in other populations (Figures S5 and S6). Specifically, HLA-DRB1*07:01 (AF = 8.1%) shows a robust binding affinity to *Corynebacterium diphtheriae*, and HLA-DRB1*08:03 (AF = 6.2%) exhibits a strong binding affinity to *Clostridium tetani* and *Bacillus anthracis* (Figure 2A). Additionally, HLA-DRB1*14:54 (AF = 2.7%) displays a notable binding affinity to *B*. *anthracis* (Figure 2B). It is important to note that there is still a subtle tendency for the adaptive HLA types to bind to peptides. The most common peptide type bound by HLA-DRB1*07:01 is “LIVS/T/KALKLI/L” (Figure S7A); the most common peptide type bound by HLA-DRB1*08:03 is “YV/KSSI/KK/NKILD” (Figure S7B); “YL/II/KK/IKKNIE” is the most common peptide type bound by HLA-DRB1*12:02 (Figure S7C); and “IKI/NS/EK/SKE/NLL/I” is the most common peptide type bound by HLA-DRB1*14:54 (Figure S7D). This preference for HLA–peptide affinity suggests that adaptive HLA types may only target one of the prevalent pathogens with the preferred peptide, and adaptation to the rest of the pathogens is simply a result of passive adaptation to a pathogen that also carries the preferred peptide. Furthermore, HLA-DQB1*03:01 and HLA-DQB1*06:01 show a pronounced affinity for specific extracellular pathogens, such as *Mycobacterium tuberculosis* and *Bordetella pertussis* (Figure 2A). The affinity of the two *HLA-DQB1* alleles for the three extracellular pathogen antigenic peptides also shows a clear preference for a particular type of amino acid sequence (Figure S8). Among the peptides bound by HLA-DQB1*03:01, there is a clear preference for the binding of the “VAAAAAAAA” peptide type (Figure S8A), whereas among the peptides bound by HLA-DQB1*06:01, the “VAAAAAAAAA” peptide type is also significantly preferred for binding (Figure S8B). This result suggests that the two HLA alleles that appear to be adapted to one of these three pathogens are also adapted to the other two.

### Antagonistic interactions between HLA alleles involved in adaptation and autoimmunity

**Figure 3:**
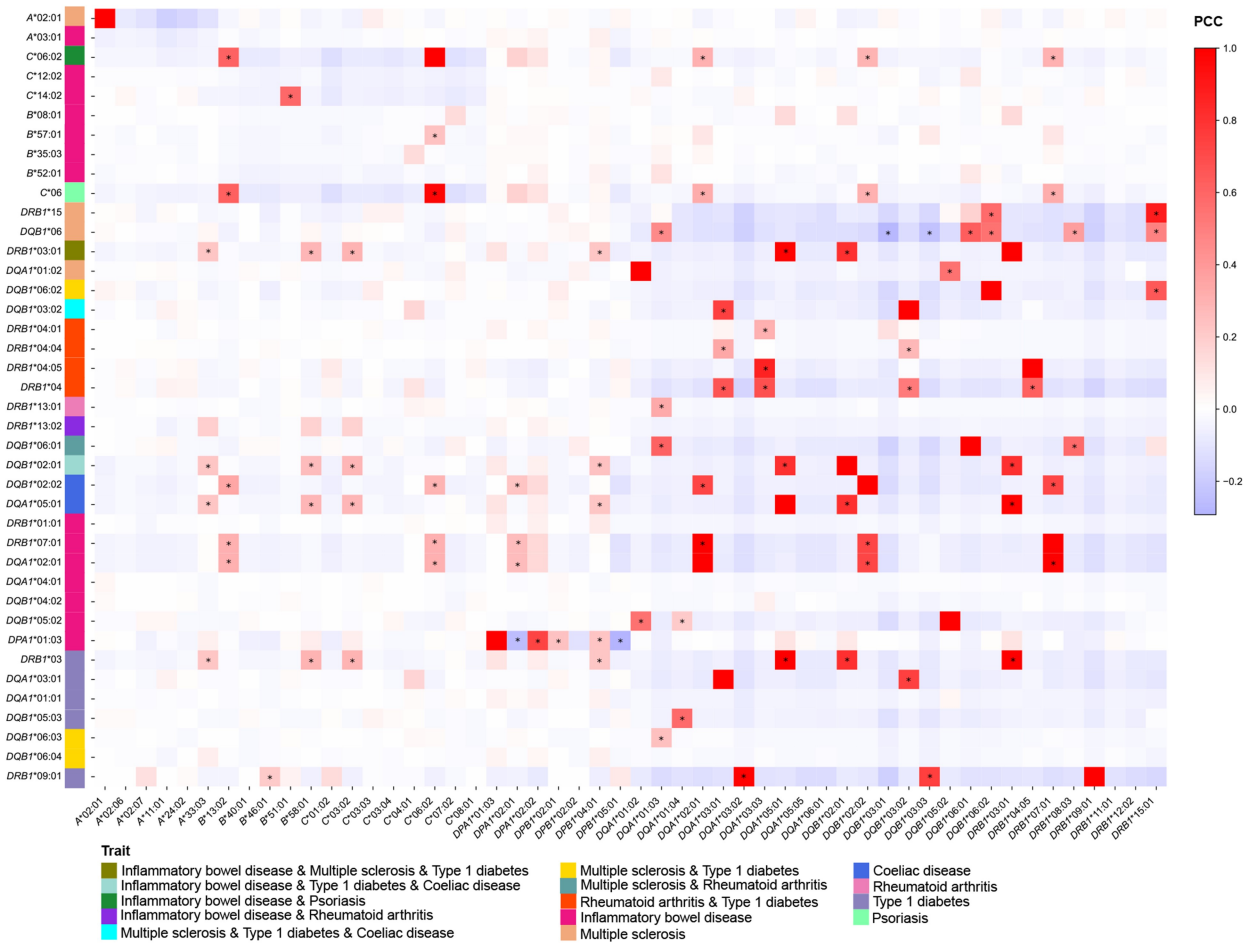
Associations between common HLA alleles in the Han Chinese population and autoimmune-susceptibility HLA alleles The X-axis represents common potential adaptive HLA alleles in the Han Chinese population, and the Y-axis represents autoimmune-susceptibility HLA alleles. Pearson correlation analyses were performed on the genotypes of the two sets of allele sets, and “*” indicates a significantly correlated gene pair (*r*^2^ > 0.2 and *P* < 0.05). PCC, Pearson correlation coefficient.

In order to investigate the genetic correlations between autoimmune diseases and pathogen adaptation in the human population, a correlation analysis was performed between the HLA alleles in the Han Chinese population and the autoimmune disease-related HLA alleles (Table S3). In general, a significant proportion of the *HLA-DRB1* alleles (HLA-DRB1*03:01, HLA-DRB1*04:05, HLA-DRB1*07:01, HLA-DRB1*09:01, HLA-DRB1*01:01, HLA-DRB1*13:01, and HLA-DRB1*13:02) as well as HLA-DQB1*06:01, which confer resistance to pathogens, have also been identified as the susceptibility alleles for numerous autoimmune diseases (Figure 2 and **Figure 3**, Figure S9). The pleiotropy of these HLA alleles provides an antagonistic balance between protection against foreign pathogens and the risk of autoimmunity [19]. In comparison to other ethnic groups, the HLA-DRB1*07:01 and HLA-DRB1*13:01 alleles in the European and African populations exert a pleiotropic effect on the correlation between susceptibility to autoimmune diseases and pathogen adaptation (Figures S10 and S11). Moreover, the high genetic linkage of HLA genes means that many pathogen-adapted HLA alleles also influence the population frequencies of autoimmune-related HLA alleles [28]. Indeed, it has been observed that over 80% of these common HLA alleles (Figure 3) and half of these low-frequency HLA alleles (Figure S9) show a significant genetic linkage to autoimmune-related HLA alleles in the Han Chinese population. In addition to the pleiotropic effects of HLA alleles, the high genetic linkage between HLA alleles represents a further significant factor contributing to the antagonistic balance between pathogen adaptation and disease risk. To illustrate, HLA-DRB1*07:01 exhibits a robust binding affinity for the antigen of *C*. *diphtheriae*, and it also shows significantly positive correlations with HLA-DQB1*02:02 (Pearson correlation coefficient $r^{2}=0.72$; the genetic distance between two HLA alleles$\;{\mathrm{DQB}1}_{cM}-{\mathrm{DRB}1}_{cM}=0.058\;$cM) and HLA-DQA1*02:01 ($r^{2}=0.99;\;{\mathrm{DRB}1}_{cM}-{\mathrm{DQA}1}_{cM}=0.014\;$cM). The former is a risk factor for coeliac disease (CD) [29], while the latter is associated with an increased risk of inflammatory bowel disease (IBD) [30] (Figure 3).

Additionally, there are complex scenarios in which HLA alleles that interact with one another exhibit varying degrees of adaptability to different pathogens. Collectively, they influence the prevalence of autoimmune-related HLA alleles in diverse directions within the population. To illustrate, HLA-DQB1*06, a risk factor for multiple sclerosis (MS) [31], has strong positive correlations with many HLA alleles associated with pathogen adaptation, including HLA-DQB1*06:01 (a subtype of HLA-DQB1*06), HLA-DRB1*08:03 ($r^{2}=0.38$; genetically linked), and HLA-DRB1*15:01 ($r^{2}=0.49$; being the strongest genetic risk factor for MS [32]). Conversely, HLA-DQB1*06 exhibits a significant negative correlation with a pathogen-adapted HLA allele HLA-DQB1*03:01 ($r^{2}=-0.28$) (Figure 3). *C*. *tetani* which produces tetanus toxin and *B*. *pertussis* which produces pertussis toxin are significant inducers of MS [33,34]. Therefore, we postulated that *C*. *tetani*, which had left adaptive genetic signatures on HLA-DRB1*08:03, and *B*. *pertussis*, which had left adaptive genetic signatures on HLA-DQB1*03:01 (Figure 2A), may have a significant impact on the genetic susceptibility to MS in the Han Chinese population in the recent evolutionary history. As an additional example, HLA-DPB1*02:01, HLA-DPB1*04:01, and HLA-DPB1*05:01 represent the alleles at the *HLA-DPB1* locus, where the first two are positively correlated with HLA-DPA1*01:03, while the latter is negatively correlated with HLA-DPA1*01:03, which is the protective factor for IBD [30] (Figure 3). IBD has been reported to be associated with infection with a range of intestinal pathogens, including *Salmonella enterica*, Epstein–Barr virus, measles virus, mumps virus, rubella virus, *Entamoeba histolytica*, and *Toxoplasma gondii* [35]. From a certain perspective, IBD has a more complex genetic structure due to the antagonistic evolution of HLA alleles and a wide range of intestinal pathogens.

### Pathogen-driven adaptation of transcriptional regulation of *HLA-C*

**Figure 4:**
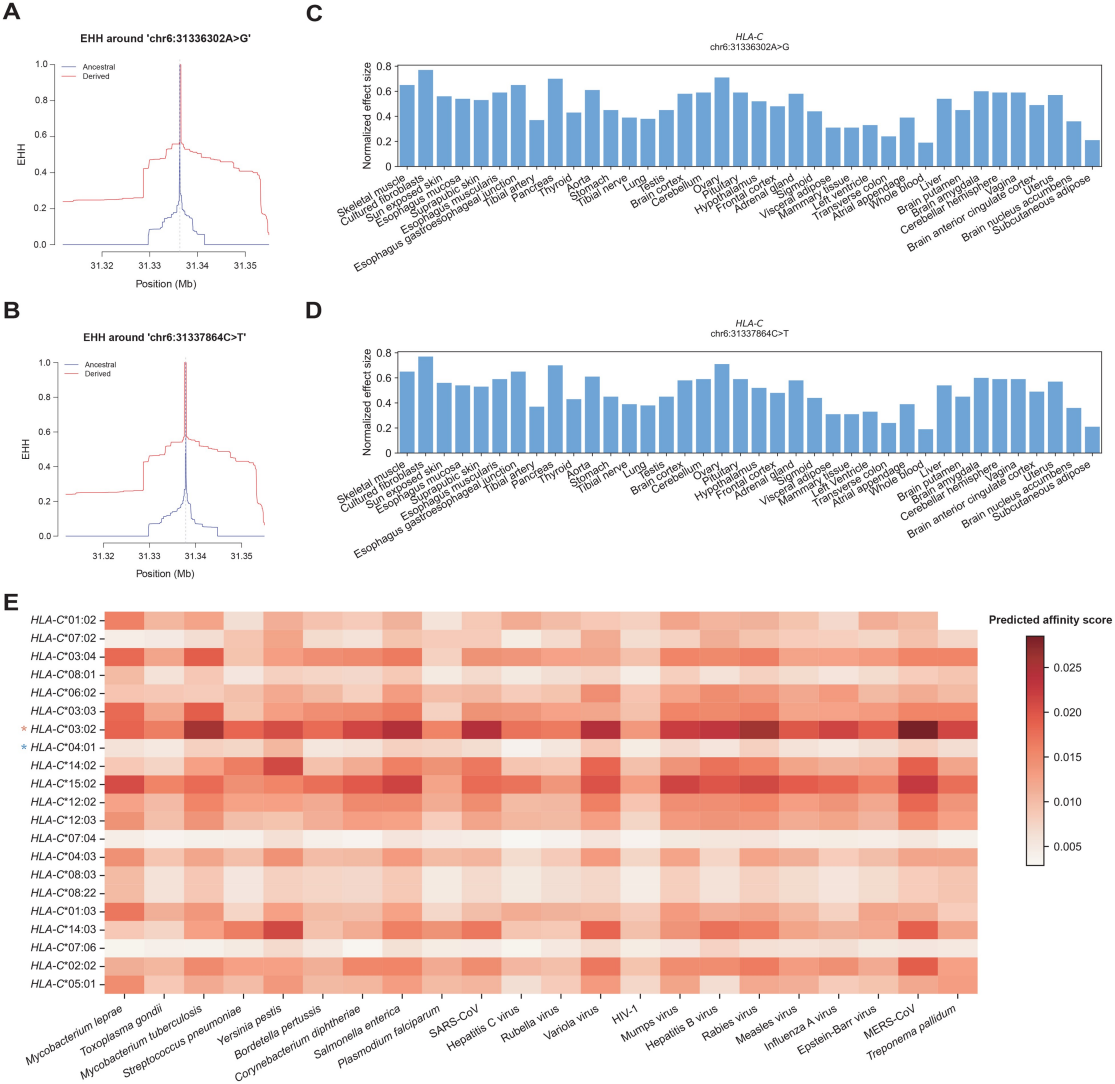
The transcriptional regulation of *HLA-C* under recent positive selection **A**. EHH plot of the focal marker chr6:31336302A>G. **B**. EHH plot of the focal marker chr6:31337864C>T. **C**. Normalized effect size of chr6:31336302A>G on the expression of *HLA-C* across tissues. **D**. Normalized effect size of chr6:31337864C>T on the expression of *HLA-C* across tissues. **E**. Binding affinity of *HLA-C* alleles with 20 pathogens that secretes intracellular antigens. Higher scores indicate stronger binding affinity. The *HLA-C* allele marked with a red star represents a significant positive correlation with the favoured regulator, while the *HLA-C* allele marked with a blue star represents a significant negative correlation with the favoured regulator. EHH, extended haplotype homozygosity.

In our work, we identified the upstream regulatory region of the *HLA-C* gene, which contains two genetically linked expression quantitative trait loci (eQTLs), chr6:31336302A>G and chr6:31337864C>T, as a target of recent positive selection based on integrated haplotype score (iHS) and singleton density score (SDS) analyses (Figures S12 and S13). The derived alleles of these selected eQTLs exhibit a significantly extended haplotype homozygosity (**Figure 4**A and B) and are characterized by a higher frequency in the East Asian population compared to the European and African populations (Figure S14). By leveraging tissue-specific gene expression data from the Genotype-Tissue Expression (GTEx) Project, we determined that the favoured alleles in question are prone to promoting the expression of *HLA-C* across a range of tissues (Figure 4C and D). This elevated immune-related transcriptional level of *HLA-C* plays an essential role in strengthening the body’s defences against pathogens. In accordance with Ohta’s nearly neutral theory of evolution, a mutation is considered nearly neutral if the selection coefficient s for the mutation meets the condition 0.2<|2Ns|<4 [36]. By tracking historical allele frequencies, it was observed that these two adaptive loci have been subject to a selection coefficient of 0.0009 (2×Ne×s=2×20000×0.0009=36>1, indicating a slightly strong selection pressure) (Figures S15 and S16). It is noteworthy that the eQTL chr6:31337864C>T has been identified as a risk genetic factor for schizophrenia, with the potential for elevated prevalence due to the pressures of pathogen-driven selection.

Furthermore, the impact of recent positive selection on the regulation of *HLA-C* alleles was examined, and it was determined that HLA-C*03:02 and HLA-C*04:01 are significantly linked with the favoured eQTL (Figure 4E). HLA-C*03:02, which has a higher allele frequency in East Asians, has been associated with a broad and potent resistance to various pathogens, especially *M*. *tuberculosis*, *S*. *enterica*, variola virus, rabies virus, and coronavirus. In contrast, HLA-C*04:01, which has the lowest allele frequency in East Asians, exhibits minimal resistance to pathogens (Figure 4E). Our findings suggest that the transcriptional regulation adjustment of HLA-C*03:02 has contributed to the enhanced general adaptability of the Han Chinese population to a range of pathogens. The affinity of HLA-C*03:02 for different pathogens is significantly different, which may be related to the specific preference of this allele for antigenic sequences. Therefore, we analyzed the motif characteristics of peptides binding to HLA-C*03:02 and found that the amino acid fragments that can be bound by this HLA allele are very diverse and do not show significant motif preferences (Figure S17), which reveals the broad spectrum of adaptation of this HLA allele to pathogens.

It is also possible that *HLA-C*’s adaptation to pathogens at the gene expression level may affect HLA alleles related to human autoimmune diseases. Although HLA-C*04:01 does not show a significant correlation with HLA alleles linked to known autoimmune diseases such as IBD, MS, type 1 diabetes (T1D), and CD, HLA-C*03:02 is associated with these diseases through genetic linkage (Figure 3). Four HLA alleles are significantly and positively correlated with HLA-C*03:02, including HLA-DRB*03:01 ($r^{2}=0.29$; susceptible to MS and T1D, protective for IBD), HLA-DQB*02:01 ($r^{2}=0.28$; susceptible to CD and T1D, protective for IBD), HLA-DQA1*05:01 ($r^{2}=0.29$; susceptible to CD), and HLA-DRB*03 ($r^{2}=0.29$; susceptible to T1D) (Figure 3; Table S2). Therefore, from the perspective of the highly linked nature of the HLA region, the adaptive selection of HLA alleles in gene expression will also affect the prevalence of autoimmune diseases in the population.

### A high-resolution HLA allele frequency database

**Figure 5:**
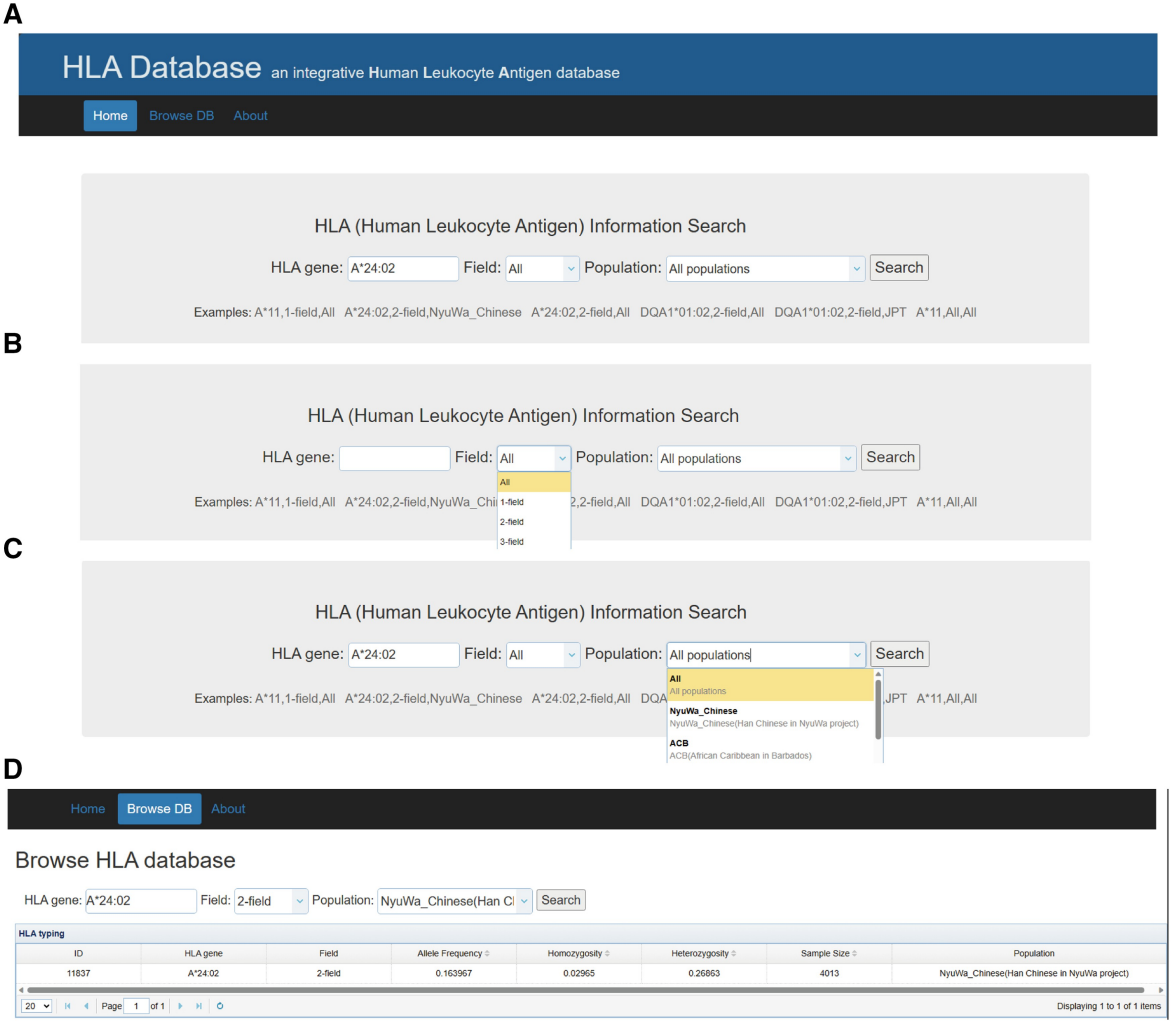
The graphic user interface of the HLA database **A**. A search bar is provided for the purpose of facilitating the input of search terms. **B**. The option for the field of HLA genotypes. **C**. The option for populations. **D**. Display of search results.

To facilitate data sharing, we developed a comprehensive HLA allele frequency database (http://bigdata.ibp.ac.cn/HLAtyping), which enables users to search for allele frequencies and homozygosity of HLA genes across various populations, with resolution ranging from serotyping level to exon-genotyping level. This database offers a user-friendly interface with an intuitive search function on its homepage (**Figure 5**A), whereby users can input the name of the HLA allele to access relevant information. To further enhance the search experience, the database has been designed to include customizable options for selecting the genotyping fields (Figure 5B) and specifying the population of interest (Figure 5C). The database provides comprehensive information for each HLA allele, including allele frequency, homozygosity, and heterozygosity (the proportion of carriers who are not homozygous) (Figure 5D). The allele frequency is a fundamental characteristic of HLA alleles within a population and is instrumental for analyzing population genetic structure, inferring demographic history, and assessing the immune adaptability to pathogenic antigens (both infectious and tumorous antigens) among human populations. The proportion of homozygous and heterozygous forms of an HLA allele provides a more detailed understanding than allele frequency alone, offering insights into the individual-level adaptation to the pathogenic environments.

## Discussion

Pathogenic microbes represent an important driving force influencing human adaptive evolution [6]. It has been widely reported that HLA alleles are associated with susceptibility to pathogens [37–42] and autoimmune diseases [18,43–47]. The theory of pathogen-driven HLA diversity has been well developed to explain the significant association between the variants of HLA alleles and infectious diseases, as well as the autoimmune diseases [18,40,48]. Fortunately, the recent availability of large-scale genome sequence data in the human population [20–22] has facilitated the investigation of evolutionary relationships between HLA and pathogens at the population level. In light of the findings on host–pathogen coevolution on HLA–peptide binding affinity from previous studies [15,16], we performed an analysis of the binding affinity between pathogen peptides and the predominant HLA types in the Han Chinese population. Our results indicate that a significant number of HLA types possess varying degrees of binding ability to numerous pathogens (Figure 2A and B, Figure S5), and are closely associated with the genetic risk of autoimmune diseases due to gene pleiotropy or genetic linkage (Figure 3).

The *HLA-DRB1* alleles exhibit notably stronger binding affinity to various pathogens than other HLA genes (Figure 2). This suggests that *HLA-DRB1* alleles have played a more substantial role in shaping the historical adaptation of human population to the intricate and varied pathogenic environment. *HLA-DRB1* plays a central role in the immune system by presenting peptides derived from extracellular proteins [49], and has the most diversity in the class II HLA genes [50]. A phylogenetic study revealed that the *HLA-DRB1* gene clusters human-specific alleles [51], suggesting that *HLA-DRB1* has been in a long-term arms race with various pathogens and may have contributed to lots of local adaptation of humans. Furthermore, in this study, the affinity between HLA and pathogens is measured by the average of the affinity scores between HLA and different peptides of pathogen, which describes the relative probability score of an HLA molecule recognizing a pathogen antigen. In light of the potential for the strong binding effects of specific antigenic peptides to be obscured (*e.g.*, by a peptide with 0.85 affinity from a pathogen and three peptides with 0.30 affinity from another pathogen, resulting in an overall affinity score of 0.9), we are endeavoring to develop more appropriate methods for measuring the affinity scores between HLA and pathogens.

Assessing the impact of pathogen adaptation on the prevalence of autoimmune diseases in human populations is an important issue. There are two major genetic factors mediating the interaction between pathogen adaptation and autoimmune diseases. One is the pleiotropy of HLA alleles. In the Finnish population, several HLA alleles have been reported to be associated with susceptibility to infectious diseases and autoimmune diseases, such as HLA-DQA1*03:01 and HLA-DQB1*03:02 [52]. Another one is the genetic linkage between nearby HLA alleles. Many of HLA-locus-based associations result from linkage disequilibrium between the HLA gene studied and other HLA genes or non-HLA genes close by [53]. In this work, the gene pleiotropy and the genetic linkage between HLA genes represent a further avenue of enquiry in our research on measuring associations between the pathogen-adapted HLA alleles and the autoimmune-related HLA alleles. However, autoimmune diseases are also the interactive result of genes and living environment, and involve confounder factors such as sex bias and infections [54]. Therefore, it is still a challenge for us to discriminate the effects of HLA alleles on the induction of autoimmunity, especially when the HLA alleles are affected by pathogen adaptation, and it is more difficult for us to quantify how the effect of specific pathogens on HLA genes will affect the occurrence and development of autoimmune diseases.

While our research, bolstered by clinical data on HIV and HCV infections [24,25], has identified a significant positive correlation between HLA–peptide binding affinity and the host’s susceptibility to infection, further clinical data encompassing a broader spectrum of pathogens are necessary to fully elucidate the extent of this association. Furthermore, further functional validation experiments have been proposed as a potential avenue for future investigation. Two aspects of interaction are worthy of consideration: (1) the interaction between antigen peptides and HLA and (2) the interaction between HLA–pathogen binding and pathogen infection efficiency. Additionally, it is important to recognize the variability in pathogen strains across different geographical regions [55–57]. Consequently, future research endeavors should prioritize the investigation of human adaptive evolution to pathogens at the strain level, taking into account the diverse nature of these pathogens and their impact on human populations.

The topic of T cell clonality is also worthy of discussion. Following the processing of extracellular antigens into peptides and subsequent complexation with surface class II HLA molecules in on professional antigen-presenting cells such as dendritic cells, these HLA–peptide complexes are presented and recognized by CD4^+^ helper T cells [58]. The clonotype of a T cell population represents a molecular description of the unique sequences required to produce the antigen specificity of the T-cell receptor (TCR), as well as the specific variable (V) and joining (J) gene segment involved in the composite rearrangements, following the completion of the selection and maturation process of T cells in the thymus [59]. This adaptive immune process represents a pivotal step in the host’s recognition of exogenous pathogens. The generation of distinct lymphocyte clones within an individual, driven by processes such as somatic mutations and V(D)J recombination, also impacts the antigen recognition. Further investigation into how these mechanisms influence lymphocyte clonality is highly warranted. This could involve utilizing high-depth whole-genome sequencing data to analyze variant allele fractions (VAFs) in lymphocytes and conducting single-cell studies to identify clonal subpopulations. Future research would take this into account, as well as other immune processes involved in the recognition of exogenous pathogens.

In future research, we intend to extend our investigation to encompass a more diverse range of populations. It is important to consider the differences in the level of medical and health development in different regions, the differences in pathogen infection rates, and the differences in the susceptibility of people to autoimmune diseases, and thus our future research aims to investigate the racial adaptation of pathogens and the racial heterogeneity of autoimmune diseases, by combining race-specific genetic data with epidemiological data.

## Materials and methods

### Data collection and processing

In this study, we utilized genome data from a total of 8278 human individuals for HLA genotyping. The dataset comprised 4013 unrelated individuals from the NyuWa Genome Project, 2504 unrelated individuals from the 1KGP (https://ftp-trace.ncbi.nlm.nih.gov/1000genomes/ftp/1000G_2504_high_coverage/), and 828 unrelated individuals from the HGDP (http://ftp.1000genomes.ebi.ac.uk/vol1/ftp/data_collections/HGDP/), as well as 116, 698, and 119 related samples from each genome project. The quality-controlled alignment files [Binary Alignment Map (BAM) format] for the 1KGP and HGDP samples were downloaded from their respective repositories, and the whole-genome sequencing data for the NyuWa Genome Project samples were processed in alignment with the methods previously described [20]. Subsequently, all reads that had been assigned to the HLA region (chr6:28,510,120–33,480,577, according to the GRCh38 assembly) were extracted for HLA genotyping. This encompasses reads that correspond to HLA genes, as well as those in an unmapped state. This approach ensures the full utilization of sequencing data information in the HLA region, thereby reducing the false positive rate of HLA genotyping. The genetic variation data employed in this study were derived from a previous work (http://bigdata.ibp.ac.cn/NyuWa_variants/http://ftp.ensembl.org/pub/release-106/fasta/ancestral_alleles/homo_sapiens_ancestor_GRCh38.tar.gz) [20]. The ancestral sequence of the human genome in GRCh38 was obtained from the Ensembl ftp site (http://ftp.ensembl.org/pub/release-106/fasta/ancestral_alleles/homo_sapiens_ancestor_GRCh38.tar.gz), and the multi-tissue eQTL data of the GTEx (v8 release) were downloaded from https://gtexportal.org/home/downloads/adult-gtex/qtl.

### HLA genotyping

The HLA typing from High-quality Dictionary (HLA-HD) software [60] (https://www.genome.med.kyoto-u.ac.jp/HLA-HD/) is a high-fidelity tool designed for high-resolution and precise HLA genotyping. In this study, HLA genes were genotyped using HLA-HD, with all reads that were potentially derived from the HLA genes realigned to the reference sequences from the Immuno Polymorphism Database-ImMunoGeneTics project/HLA Database (IPD-IMGT/HLA, release 3.45.0). To evaluate the accuracy of HLA genotyping, we employed the “golden sets” of HLA data typed by PCR-Sequence-Based Typing (PCR-SBT) technique from 1206 individuals from the 1KGP [7] (https://ftp.1000genomes.ebi.ac.uk/vol1/ftp/technical/working/20140725_hla_genotypes/). The accuracy for each HLA gene was calculated by summing the dosage of all correctly inferred HLA alleles across all individuals (n) and dividing by the total number of observations (2n) [13]. That is,


(1) $$\mathrm{Accuracy}(g)=\frac{\sum_{i}^{n}D_{i}(A_{1i,g})+\sum_{i}^{n}D_{i}(A_{2i,g})}{2n}$$

where Accuracy(g) represents the accuracy at a classical HLA gene (*e.g.*, *HLA-C*). $D_{i}$ represents the inferred dosage of an allele in individual i, and alleles $A_{1i,g}$ and $A_{2i,g}$ represent the true (PCR-SBT-based “golden sets”) HLA types for an individual i.

### HLA–peptide binding affinity analysis

In our investigation of the adaptability of HLA alleles to various epidemic pathogens (Table S2), we established a quantitative measure to define the affinity between an HLA molecule and a pathogen antigen as follows:


(2) $$A=\frac{\sum_{i=1}^{N}{\mathrm{Score}\_EL}_{i}}{N}$$

where *N* denotes the total count of peptides derived from the antigen, and ${\mathrm{Score}\_EL}_{i}$ signifies the predicted binding score (predicted by NetMHCpan-4.1 and NetMHCIIpan-4.0 [61]) for the HLA–peptide pair that has significant binding affinity, comprising strong binders if the binding affinity ranking is in the top 0.5% and weak binders if the binding affinity ranking is in the top 2%. The quantification of HLA–peptide binding affinity is a well-established approach for prognosing the pathogen resistance of individual hosts [62]. Consequently, the metric A serves as a descriptive tool to articulate the likelihood of a host’s immune system effectively identifying and responding to pathogenic threats.

### Correlation analysis of HLA–peptide binding affinity and pathogen resistance

To evaluate the relationship between HLA–peptide binding affinity and pathogen resistance, we employed the mean value of predicted binding scores for HLA interactions with a pathogen’s peptides as an evaluative metric. To investigate the clinical adaptation of HIV and HCV infections to host HLA genotypes, we calculated the predicted mean binding scores for the engagement of HLA molecules with pathogenic peptides, drawing on two previous studies that had explored this topic [24,25]. Subsequently, we compared these scores with various measures of pathogen resistance, including viral setpoint, progression of viral disease, and the status of viral RNA level, in order to ascertain the efficacy of HLA genes across different levels of binding affinity.

### Reconstruction of HLA reference panel

Following the established pipeline for constructing an HLA reference panel (https://github.com/immunogenomics/HLA-TAPAS) [13], we integrated HLA genotypes with haplotype reference panels that were delineated from single nucleotide polymorphism (SNPs) and insertions and deletions (indels) within the HLA region. The initial step entailed the recapturing of variants missed in the initial analysis due to the high-degree polymorphism of HLA genes. This was accomplished by realigning the sequences of the genotyped HLA genes to the GRCh38 reference assembly. Subsequently, variants with minor allele frequency (MAF) > 0.001 within the MHC region were extracted from three haplotype reference panels of the NyuWa Han Chinese population, the 1KGP, and the HGDP. In the final step, the HLA genotypes were integrated into the aforementioned haplotype reference panels, after which the haplotypes were re-phased using Segmented Haplotype Estimation and Imputation Tools version 4 (SHAPEIT4) [63].

### Selection analysis of HLA regulators

To detect adaptive selection within HLA regulatory regions, we systematically computed the SDS and iHS statistics for genome-wide detection of recent positive selections and the Beta statistics for long-term balancing selections in the NyuWa Han Chinese population [64–67]. In order to ensure the reliability of the results, we applied rigorous criteria to common variants, defined by MAF ≥ 0.05. Subsequently, the aforementioned common variants were employed in the calculation of SDS, iHS, and Beta statistics. Statistics with a false discover rate (FDR) adjusted using the Benjamini–Hochberg procedure of less than 0.05 (|SDS| ≥ 3.857, |iHS| ≥ 4.234, and Beta ≥ 8.41) are considered to indicate significant selection signals. To evaluate the robustness of potential selective sweeps or balancing selection events, a sliding window approach was employed, with each segment comprising 100 SNPs and a step size of 50 SNPs. Intervals with a higher proportion of significant signals within this framework were considered as indicative of more robust selective events. To substantiate the implications of these selection signals on phenotypic variation, we leveraged eQTL data from the GTEx project, which provided evidence for alterations in gene expression at the transcriptional level [68]. By combining selection statistics with functional genomic data, we inferred the potential fitness effects of adaptive regulatory changes in the HLA region.

### Allele frequency trajectory computation

An approximate full-likelihood method [69] was employed to deduce the selection coefficients and allele frequency trajectories for the alleles under selection. In this analysis, our focus was on a 5-kb haploblock, characterized by infrequent recombination, which encompasses the two adaptive sites of interest. This region was selected for the reconstruction of allele frequency trajectories over the last 1000 generations, with the stipulation that the indels within the haploblock were not considered. In preparation for inferring the allele frequency trajectories, the Relate software [70] was employed to estimate the genealogical and population history of the haploblock. For the Han Chinese population, the effective population size was set to 20,000, the mutation rate was set to 1.25E−8, the years per generation was set to 28, and the number of sampling times of branch lengths was set to 100. All other parameters were configured according to their default settings.

## Ethical statement

This study was approved by the Medical Research Ethics Committee of Institute of Biophysics, Chinese Academy of Sciences (Approval No. IBP-2016-XT-1). All participants provided written informed consent for sample collection for genome studies conducted by Chinese Academy of Sciences. Eligible participants included both patients and healthy individuals aged 30–70 years with full decision-making capacity. Participants voluntarily donated blood samples, and provided clinical treatment information. All personal information was kept confidential, and participants could refuse to participate in sample donation or withdraw at any time.

## Supplementary Material

qzaf038_Supplementary_Data

## Data Availability

The DNA sequencing data of NyuWa samples have been deposited in the Genome Sequence Archive for Human [71] at the National Genomics Data Center (NGDC), China National Center for Bioinformation (CNCB) (GSA-Human: HRA004185), and are publicly accessible at https://ngdc.cncb.ac.cn/gsa-human/.

## CRediT author statement

**Shuai Liu:** Conceptualization, Investigation, Methodology, Formal analysis, Visualization, Writing – original draft, Writing – review & editing. **Yanyan Li:** Conceptualization, Investigation, Methodology, Formal analysis, Visualization, Writing – original draft, Writing – review & editing. **Tingrui Song:** Conceptualization, Methodology, Formal analysis, Visualization, Writing – original draft, Writing – review & editing. **Jingjing Zhang:** Investigation, Methodology, Formal analysis. **Peng Zhang:** Data curation, Methodology, Formal analysis. **Huaxia Luo:** Methodology, Formal analysis. **Sijia Zhang:** Data curation. **Yiwei Niu:** Data curation. **Tao Xu:** Conceptualization, Funding acquisition, Project administration. **Shunmin He:** Conceptualization, Funding acquisition, Project administration, Writing – review & editing. All authors have read and approved the final manuscript.
