# Supplementary material for: Pathogen Adaptation of HLA Alleles and Its Correlation with Autoimmune Diseases in the Han Chinese
Source: Genomics Proteomics Bioinformatics. 2025 Apr 29;23(2):qzaf038. doi: 10.1093/gpbjnl/qzaf038 (PMC12368854; doi:10.1093/gpbjnl/qzaf038)
Supplement: qzaf038_Supplementary_Data [file qzaf038_supplementary_data.zip › supplementary material captions.docx]

**Supplementary material**

**Figure S1　Major HLA Alleles in the Han Chinese**

**A.** NyuWa Han Chinese. **B.** East Asian. **C.** European. **D.** African.

**Figure S2　Relationship between HIV-associated HLA genes and HIV invasion ability and *in vivo* reproductive ability**

Viral setpoint is the time at which plasma viraemia settles to a relatively stable level (within approximately 3–6 months of the onset of HIV infection). Viral setpoint is strongly predictive of both how quickly HIV infection will progress and the risk of HIV transmission. Progression is the rate of disease progression. Scaled score EL is the mean of binding affinity of HLA molecule with peptides of a pathogen. HIV, human immunodeficiency virus; EL, Mass-Spectrometry Eluted Ligands.

**Figure S3　Relationship between HCV-associated HLA genes and the RNA status of HCV**

HCV RNA status means HCV RNA level *in vivo*. HCV, hepatitis C virus.

**Figure S4　The binding affinity between the epidemic pathogens and the rare HLA types in the Han Chinese population**

The pathogens in the yellow block are virus, the pathogens in the pink block are bacteria, and the pathogens in the blue block are parasites. The higher score means higher affinity.

**Figure S5　The binding affinity between the epidemic pathogens and the common HLA types in the European population and the African population**

**A.** European**. B.** African. The pathogens in the yellow block are virus, the pathogens in the pink block are bacteria, and the pathogens in the blue block are parasites. The higher score means higher affinity.

**Figure S6　The binding affinity between the epidemic pathogens and the low frequency and rare HLA types in the European population and the African population**

**A.** European**. B.** African. The pathogens in the yellow block are virus, the pathogens in the pink block are bacteria, and the pathogens in the blue block are parasites. The higher score means higher affinity.

**Figure S7　Affinity preference of three *HLA-DRB1* alleles for pathogen peptides**

**A.** Peptide-binding preference of HLA-DRB1*07:01 for *Corynebacterium diphtheriae*. **B.** Peptide-binding preference of HLA-DRB1*08:03 for *Clostridium tetani*. **C.** Peptide-binding preference of HLA-DRB1*08:03 for *Bacillus anthracis*. **D.** Peptide-binding preference of HLA-DRB1*14:54 for *Bacillus anthracis*.

**Figure S8　Affinity preference of two *HLA-DQB1* alleles for pathogen peptides**

**A.** HLA-DQB1*03:01. **B.** HLA-DQB1*06:01. Here mainly revolves 3 pathogens, including *Bordetella pertussis*, *Mycobacterium tuberculosis*, and *Corynebacterium diphtheriae*.

**Figure S9　Correlation between the low-frequency HLA alleles in the Han Chinese and the autoimmune susceptibility HLA alleles**

The horizontal axis represents common potential adaptive HLA alleles in the Han Chinese population, and the vertical axis represents autoimmune susceptibility HLA alleles. Pearson correlation tests were performed on the genotypes of the two sets of gene sets, “*” indicates significantly correlated gene pairs (r^2^ > 0.2 and *P* < 0.05).

**Figure S10　Correlation between the common HLA alleles and the autoimmune susceptibility HLA alleles in the European population and the African population**

**A.** European**. B.** African. The horizontal axis represents common potential adaptive HLA alleles in the Han Chinese population, and the vertical axis represents autoimmune susceptibility HLA alleles. Pearson correlation tests were performed on the genotypes of the two sets of gene sets, “*” indicates significantly correlated gene pairs (r^2^ > 0.2 and *P* < 0.05).

**Figure S11　Correlation between the low-frequency and rare HLA alleles and the autoimmune susceptibility HLA alleles in the European population and the African population**

**A.** European**. B.** African. The horizontal axis represents common potential adaptive HLA alleles in the Han Chinese population, and the vertical axis represents autoimmune susceptibility HLA alleles. Pearson correlation tests were performed on the genotypes of the two sets of gene sets, “*” indicates significantly correlated gene pairs (r^2^ > 0.2 and *P* < 0.05).

**Figure S12　The adaptive selection signals in the MHC region**

The dashed line marked the significant level of statistics. SDS, singleton density score; iHS, integrated haplotype score; FDR, false discover rate; MHC, major histocompatibility complex.

**Figure S13　The adaptive selection signals in 6p21.33**

**A.** Genetic diversity. **B.** Recombination rate. **C.** Proportion of Beta1 greater than 8.41 in a 100 snp window. **D.** Proportion of |iHS| greater than 4.234 in a 100 snp window.

**Figure S14　The allele frequencies of the adaptive variants in East Asian, European, and African from 1KGP**

**Figure S15　Allele frequency trajectories of 6:31336302G in recent 1000 generations**

**Figure S16　Allele frequency trajectories of 6:31337864T in recent 1000 generations**

**Figure S17　Affinity preference of HLA-C*03:02 for all pathogen peptides**

**A.** Sequence characteristics of all pathogen antigen peptides that have the ability to bind to HLA-C*03:02. **B.** Sequence characteristics of all pathogen antigen peptides with strong binding ability to HLA-C*03:02. **C.** Sequence characteristics of all pathogen antigen peptides with weak binding ability to HLA-C*03:02.

**Table S1　Adaptive HLA frequencies and pathogen prevalence in the Chinese, European, and African populations**

**Table S2　Pathogenic antigen data information**

**Table S3　Autoimmune diseases related data information**
