# Supplementary material for: Pathogen Adaptation of HLA Alleles and Its Correlation with Autoimmune Diseases in the Han Chinese
Source: Genomics Proteomics Bioinformatics. 2025 Apr 29;23(2):qzaf038. doi: 10.1093/gpbjnl/qzaf038 (PMC12368854; doi:10.1093/gpbjnl/qzaf038)
Supplement: qzaf038_Supplementary_Data [file qzaf038_supplementary_data.zip › Table S1.docx]

**Table S1 Adaptive HLA frequencies and pathogen prevalence in the Chinese, European, and African populations**

| **Region** | **Pathogen** | **Prevalence** | **HLA** | **Allele frequency** |
| --- | --- | --- | --- | --- |
| China | *Mycobacterium tuberculosis* | 0.01396276% | *HLA-DQB1**03:01 | 0.219038 |
| Europe | *Mycobacterium tuberculosis* | 0.00545723% | *HLA-DQB1**03:01 | 0.16998 |
| Africa | *Mycobacterium tuberculosis* | 0.02909728% | *HLA-DQB1**03:01 | 0.079425 |
| China | HIV-1 | 0.00071679% | *HLA-B**13:02 | 0.050087 |
| Europe | HIV-1 | 0.00486063% | *HLA-B**13:02 | 0.026839 |
| Africa | HIV-1 | 0.01273640% | *HLA-B**13:02 | 0.01059 |
| China | *Corynebacterium diphtheriae* | 0.00000177% | *HLA-DRB1**07:01 | 0.081111 |
| Europe | *Corynebacterium diphtheriae* | 0.00000043% | *HLA-DRB1**07:01 | 0.140159 |
| Africa | *Corynebacterium diphtheriae* | 0.00014464% | *HLA-DRB1**07:01 | 0.066566 |
| China | *Bordetella pertussis* | 0.00304545% | *HLA-DQB1**03:01 | 0.219038 |
| Europe | *Bordetella pertussis* | 0.00128790% | *HLA-DQB1**03:01 | 0.16998 |
| Africa | *Bordetella pertussis* | 0.05378022% | *HLA-DQB1**03:01 | 0.079425 |
| China | *Mycobacterium leprae* | 0.00001176% | *HLA-B**13:02 | 0.050087 |
| Europe | *Mycobacterium leprae* | 0.00000000% | *HLA-B**13:02 | 0.026839 |
| Africa | *Mycobacterium leprae* | 0.00008933% | *HLA-B**13:02 | 0.01059 |
| China | SARS-CoV-2 | 0.00136936% | *HLA-C**03:02 | 0.063543 |
| Europe | SARS-CoV-2 | 7.57396252% | *HLA-C**03:02 | 0.002982 |
| Africa | SARS-CoV-2 | 7.39087985% | *HLA-C**03:02 | 0.029501 |

*Note*: The prevalence data for infectious diseases from the Global Burden of Disease (https://www.healthdata.org/research-analysis/gbd)**.**
