# Supplementary material for: Pathogen Adaptation of HLA Alleles and Its Correlation with Autoimmune Diseases in the Han Chinese
Source: Genomics Proteomics Bioinformatics. 2025 Apr 29;23(2):qzaf038. doi: 10.1093/gpbjnl/qzaf038 (PMC12368854; doi:10.1093/gpbjnl/qzaf038)
Supplement: qzaf038_Supplementary_Data [file qzaf038_supplementary_data.zip › Table S2.docx]

**Table S2 Pathogenic antigen data information**

| **Categories** | **Pathogens** | **Antigens (NCBI ID or UniProt ID)** |
| --- | --- | --- |
| Intracellular | Hepatitis C virus | UP000000518 |
| Intracellular | Rubella virus | UP000000571 |
| Intracellular | Variola virus | UP000002060 |
| Intracellular | HIV-1 | UP000002241 |
| Intracellular | Mumps virus | UP000002331 |
| Intracellular | Hepatitis B virus | UP000007930 |
| Intracellular | Rabies virus | UP000008649 |
| Intracellular | Measles virus | UP000008699 |
| Intracellular | Influenza A virus | UP000009255 |
| Intracellular | Epstein–Barr virus | UP000153037 |
| Intracellular | MERS-CoV | UP000171868 |
| Intracellular | SARS-CoV | UP000000354 |
| Intracellular | SARS-CoV2 | UP000464024 |
| Intracellular–Extracellular | *Treponema pallidum* | AAA75016, AAA27481, AAA27477, AAA27472, AAC45732 |
| Intracellular–Extracellular | *Mycobacterium leprae* | NP_302372, NP_301968, NP_301879, NP_301372, CAR70980 |
| Intracellular–Extracellular | *Toxoplasma gondii* | EPT30382, EPT30138, EPT30276, EPT29989, EPT26499, EPT27242, EPT26403, EPT30357, EPT29845 |
| Intracellular–Extracellular | *Mycobacterium tuberculosis* | NP_218391, YP_177853, YP_177893, NP_216548, NP_218393, YP_178022, NP_215554, NP_218321, NP_214989, NP_216495 |
| Intracellular–Extracellular | *Streptococcus pneumoniae* | NP_357715, NP_359586, NP_359346, NP_358461, NP_359024, NP_358176, NP_359129, NP_359087, NP_358155, NP_358175 |
| Intracellular–Extracellular | *Yersinia pestis* | NP_395430, NP_395429, NP_395427, NP_395165, NP_395166, NP_395143 |
| Intracellular–Extracellular | *Bordetella pertussis* | NP_882282, NP_882283, NP_882286, NP_882284, NP_882285, NP_879898, NP_880302, NP_880571, NP_879839, NP_881965 |
| Intracellular–Extracellular | *Corynebacterium diphtheriae* | NP_938615 |
| Intracellular–Extracellular | *Salmonella enterica* | NP_456106, NP_456107, NP_456109, NP_456110, NP_456114, NP_456134, NP_456135, NP_456136, NP_458730, NP_458731, NP_458734, NP_458732, NP_458733, NP_458738, NP_458739, NP_458741, NP_458735 |
| Intracellular–Extracellular | *Plasmodium falciparum* | XP_001348275, XP_001348247, XP_001350083, XP_002809051, XP_001349749, XP_001350088, XP_001349859, XP_001350569, XP_001350410, XP_001348153, XP_001348015, XP_001347895, XP_001347636, XP_001347630, XP_001347629, XP_001352222, XP_001352170, XP_001349336, XP_002808637, XP_001349578 |
| Extracellular | *Clostridium tetani* | NP_783831, YP_008774065, NP_782184, NP_780878, NP_781182 |
| Extracellular | *Vibrio cholera* | NP_231099, NP_231100, NP_231102, NP_231104 |
| Extracellular | *Bacillus anthracis* | UP000000594 |
| Extracellular | *Entamoeba histolytica* | XP_655241, EAL50306, EAL50995, XP_652992, AAC72364, XP_657050, XP_649161, AAA29100, XP_650725, XP_648032 |
| Extracellular | *Giardia lamblia* | XP_001704065, XP_001705852, XP_001705828, XP_001703888, XP_001705844, XP_001705829, XP_001705785, XP_001705784, XP_001705783, XP_001703933, XP_001703925, XP_001703844, XP_001703931 |
| Extracellular | *Trichinella spiralis* | CAA73574, EFV52545, ACV51809, EFV53657 |
| Extracellular | *Trichomonas vaginalis* | XP_001292151, XP_001313891, XP_001284871, XP_001580136, XP_001582296, XP_001299798, XP_001584317, XP_001327241, XP_001325298, XP_001303631, XP_001316801, XP_001326883, XP_001301868 |
| Extracellular | *Schistosoma mansoni* | CCD74732, CCD75328, CCD75626, CCD75625, CCD75627, CCD80234, CCD77656, CCD77655 |

*Note*: NCBI, National Center for Biotechnology Information; ID, identity document; UniProt, Universal Protein; HIV-1, human immunodeficiency virus type 1; MERS-CoV, Middle East respiratory syndrome coronavirus; SARS-CoV, severe acute respiratory syndrome coronavirus; SARS-CoV2, severe acute respiratory syndrome coronavirus 2.
