# Supplementary material for: Pathogen Adaptation of HLA Alleles and Its Correlation with Autoimmune Diseases in the Han Chinese
Source: Genomics Proteomics Bioinformatics. 2025 Apr 29;23(2):qzaf038. doi: 10.1093/gpbjnl/qzaf038 (PMC12368854; doi:10.1093/gpbjnl/qzaf038)
Supplement: qzaf038_Supplementary_Data [file qzaf038_supplementary_data.zip › Table S3.docx]

**Table S3 Autoimmune diseases related data information**

| **Traits** | **Related HLA** | **Types** | **Refs.** |
| --- | --- | --- | --- |
| Multiple sclerosis | *HLA-DRB1**15 | Risk | [1] |
| Multiple sclerosis | *HLA-DQB1**06 | Risk | [1] |
| Multiple sclerosis | *HLA-B**03:01 | Risk | [2] |
| Multiple sclerosis | *HLA-DRB1**13:03 | Risk | [2] |
| Multiple sclerosis | *HLA-DRB1**03:01 | Risk | [2] |
| Multiple sclerosis | *HLA-DRB1**08:01 | Risk | [2] |
| Multiple sclerosis | *HLA-DQA1**01:02 | Risk | [2] |
| Multiple sclerosis | *HLA-DQB1**06:02 | Risk | [2] |
| Multiple sclerosis | *HLA-DQB1**03:02 | Risk | [2] |
| Multiple sclerosis | *HLA-A**02:01 | Protective | [2] |
| Rheumatoid arthritis | *HLA-DRB1**04:01 | Risk | [3] |
| Rheumatoid arthritis | *HLA-DRB1**04:04 | Risk | [3] |
| Rheumatoid arthritis | *HLA-DRB1**04:05 | Risk | [3] |
| Rheumatoid arthritis | *HLA-DRB1**04:10 | Risk | [3] |
| Rheumatoid arthritis | *HLA-DRB1**04 | Risk | [4] |
| Rheumatoid arthritis | *HLA-DRB1**13:01 | Protective | [5] |
| Rheumatoid arthritis | *HLA-DRB1**13:02 | Protective | [5] |
| Rheumatoid arthritis | *HLA-DQB1**06:01 | Risk | [5] |
| Coeliac disease | *HLA-DQB1**02:01 | Risk | [6] |
| Coeliac disease | *HLA-DQB1**02:02 | Risk | [6] |
| Coeliac disease | *HLA-DQB1**03:02 | Risk | [6,7] |
| Coeliac disease | *HLA-DQB1**03:05 | Risk | [7] |
| Coeliac disease | *HLA-DQA1**05:01 | Risk | [8] |
| Inflammatory bowel diseases | *HLA-A**03:01 | Risk | [9] |
| Inflammatory bowel diseases | *HLA-C**06:02 | Risk | [9] |
| Inflammatory bowel diseases | *HLA-C**08:02 | Risk | [9] |
| Inflammatory bowel diseases | *HLA-C**12:02 | Risk | [9] |
| Inflammatory bowel diseases | *HLA-C**14:02 | Risk | [9] |
| Inflammatory bowel diseases | *HLA-B**08:01 | Protective | [9] |
| Inflammatory bowel diseases | *HLA-B**57:01 | Risk | [9] |
| Inflammatory bowel diseases | *HLA-B**14:02 | Risk | [9] |
| Inflammatory bowel diseases | *HLA-B**35:03 | Protective | [9] |
| Inflammatory bowel diseases | *HLA-B**52:01 | Risk | [9] |
| Inflammatory bowel diseases | *HLA-B**35:02 | Risk | [9] |
| Inflammatory bowel diseases | *HLA-DRB1**01:03 | Risk | [9] |
| Inflammatory bowel diseases | *HLA-DRB1**01:01 | Protective | [9] |
| Inflammatory bowel diseases | *HLA-DRB1**03:01 | Protective | [9] |
| Inflammatory bowel diseases | *HLA-DRB1**07:01 | Risk | [9] |
| Inflammatory bowel diseases | *HLA-DRB1**08:01 | Risk | [9] |
| Inflammatory bowel diseases | *HLA-DRB1**16:01 | Protective | [9] |
| Inflammatory bowel diseases | *HLA-DRB1**13:02 | Risk | [9] |
| Inflammatory bowel diseases | *HLA-DQA1**02:01 | Risk | [9] |
| Inflammatory bowel diseases | *HLA-DQA1**04:01 | Risk | [9] |
| Inflammatory bowel diseases | *HLA-DQB1**02:01 | Protective | [9] |
| Inflammatory bowel diseases | *HLA-DQB1**04:02 | Risk | [9] |
| Inflammatory bowel diseases | *HLA-DQB1**05:02 | Protective | [9] |
| Inflammatory bowel diseases | *HLA-DPA1**01:03 | Protective | [9] |
| Psoriasis | *HLA-C**06 | Risk | [10] |
| Type 1 diabetes | *HLA-DRB1**03 | Risk | [11] |
| Type 1 diabetes | *HLA-DRB1**04 | Risk | [11] |
| Type 1 diabetes | *HLA-DQB1**03:02 | Risk | [12] |
| Type 1 diabetes | *HLA-DQB1**02:01 | Risk | [12] |
| Type 1 diabetes | *HLA-DQA1**03:01 | Risk | [12] |
| Type 1 diabetes | *HLA-DQA1**01:01 | Protective | [12] |
| Type 1 diabetes | *HLA-DQB1**05:03 | Protective | [12] |
| Type 1 diabetes | *HLA-DQB1**06:02 | Protective | [12] |
| Type 1 diabetes | *HLA-DQB1**06:03 | Protective | [12] |
| Type 1 diabetes | *HLA-DQB1**06:04 | Protective | [12] |
| Type 1 diabetes | *HLA-DRB1**09:01 | Risk | [13] |
| Type 1 diabetes | *HLA-DRB1**04:05 | Risk | [13] |
| Type 1 diabetes | *HLA-DRB1**15:03 | Protective | [13] |

*Note*: HLA, human leukocyte antigen.

[1] De Silvestri A, Capittini C, Mallucci G, Bergamaschi R, Rebuffi C, Pasi A, et al. The involvement of HLA class II alleles in multiple sclerosis: a systematic review with meta-analysis. Dis Markers 2019;2019:1409069.

[2] Martin R, Sospedra M, Eiermann T, Olsson T. Multiple sclerosis: doubling down on MHC. Trends Genet 2021;37:784–97.

[3] Yang M, Kuang X, Li J, Pan Y, Tan M, Lu B, et al. Meta-analysis of the association of HLA-DRB1 with rheumatoid arthritis in Chinese populations. BMC Musculoskelet Disord 2013;14:307.

[4] Alsaeid K, Alawadhi A, Al-Saeed O, Haider MZ. Human leukocyte antigen DRB1*04 is associated with rheumatoid arthritis in Kuwaiti patients. Joint Bone Spine 2006;73:62–5.

[5] Furukawa H, Oka S, Shimada K, Hashimoto A, Tohma S. Human leukocyte antigen polymorphisms and personalized medicine for rheumatoid arthritis. J Hum Genet 2015;60:691–6.

[6] Gualandris F, Castellani L, Falanga A. The association of HLA-DQ2 with celiac disease. In: Chai J, editors. Celiac Dis. London: IntechOpen 2021;1–26.

[7] Abadie V, Kim SM, Lejeune T, Palanski BA, Ernest JD, Tastet O, et al. IL-15, gluten and HLA-DQ8 drive tissue destruction in coeliac disease. Nature 2020;578:600–4.

[8] Espino L, Núñez C. The HLA complex and coeliac disease. Int Rev Cell Mol Biol. 2021:358:47–83.

[9] Goyette P, Boucher G, Mallon D, Ellinghaus E, Jostins L, Huang H, et al. High-density mapping of the MHC identifies a shared role for HLA-DRB1*01:03 in inflammatory bowel diseases and heterozygous advantage in ulcerative colitis. Nat Genet 2015;47:172–9.

[10] Nestle FO, Kaplan DH, Barker J. Psoriasis. N Engl J Med 2009;361:496–509.

[11] Aly TA, Ide A, Jahromi MM, Barker JM, Fernando MS, Babu SR, et al. Extreme genetic risk for type 1A diabetes. Proc Natl Acad Sci U S A 2006;103:14074–9.

[12] Hamzeh AR, Nair P, Al-Khaja N, Al Ali MT. Association of HLA-DQA1 and -DQB1 alleles with type I diabetes in Arabs: a meta-analyses. Tissue Antigens 2015;86:21–7.

[13] Noble JA, Besançon S, Sidibé AT, Rozemuller EH, Rijkers M, Dadkhodaie F, et al. Complete HLA genotyping of type 1 diabetes patients and controls from Mali reveals both expected and novel disease associations. HLA 2024;103:e15319.

[14] Goulder PJ, Watkins DI. Impact of MHC class I diversity on immune control of immunodeficiency virus replication. Nat Rev Immunol 2008;8:619–30.
