# Supplementary figures and images for: Pathogen Adaptation of HLA Alleles and Its Correlation with Autoimmune Diseases in the Han Chinese
Source: Genomics Proteomics Bioinformatics. 2025 Apr 29;23(2):qzaf038. doi: 10.1093/gpbjnl/qzaf038 (PMC12368854; doi:10.1093/gpbjnl/qzaf038)

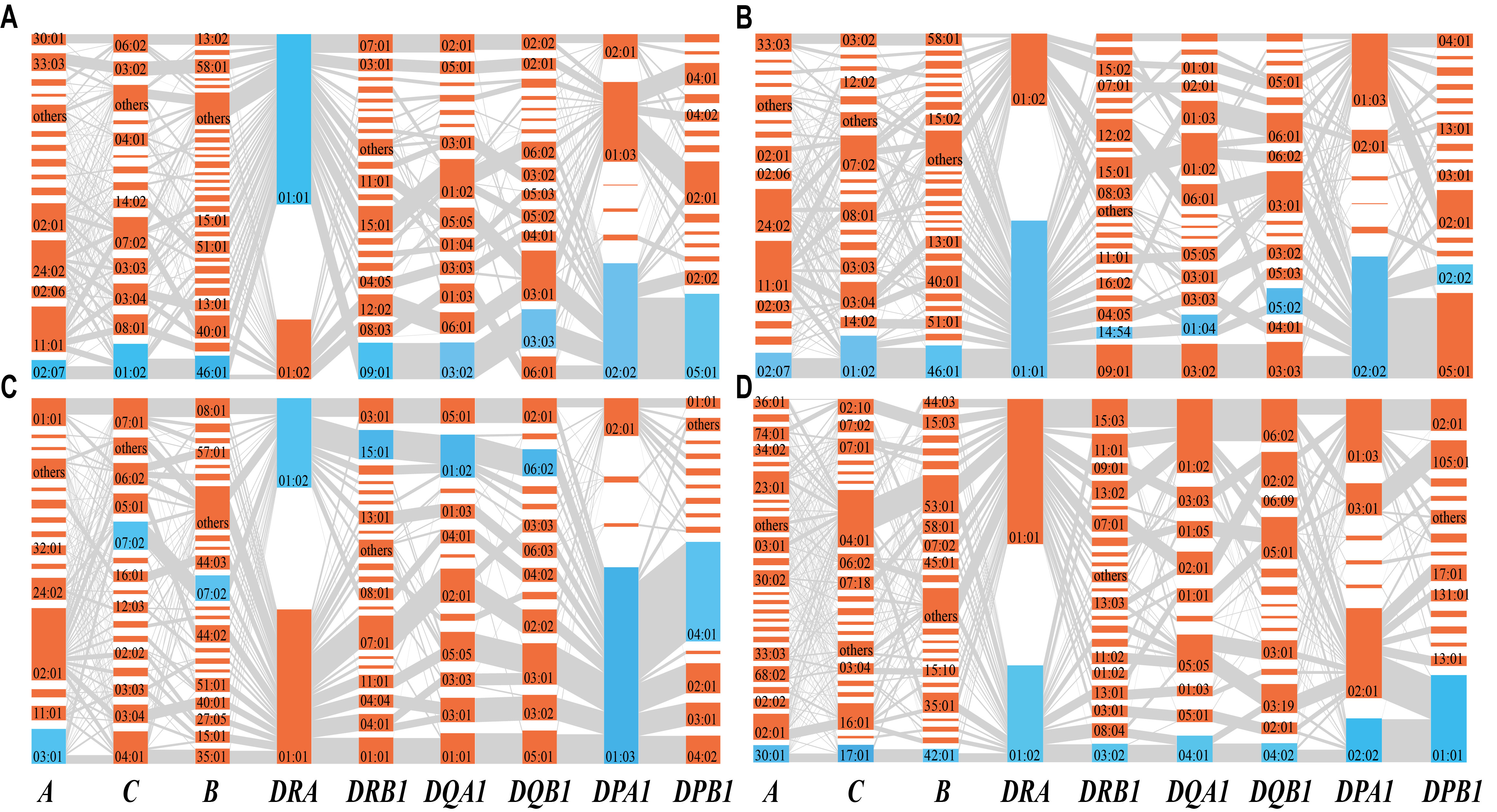

Supplement: qzaf038_Supplementary_Data [file qzaf038_supplementary_data.zip › FigureS1.jpg]

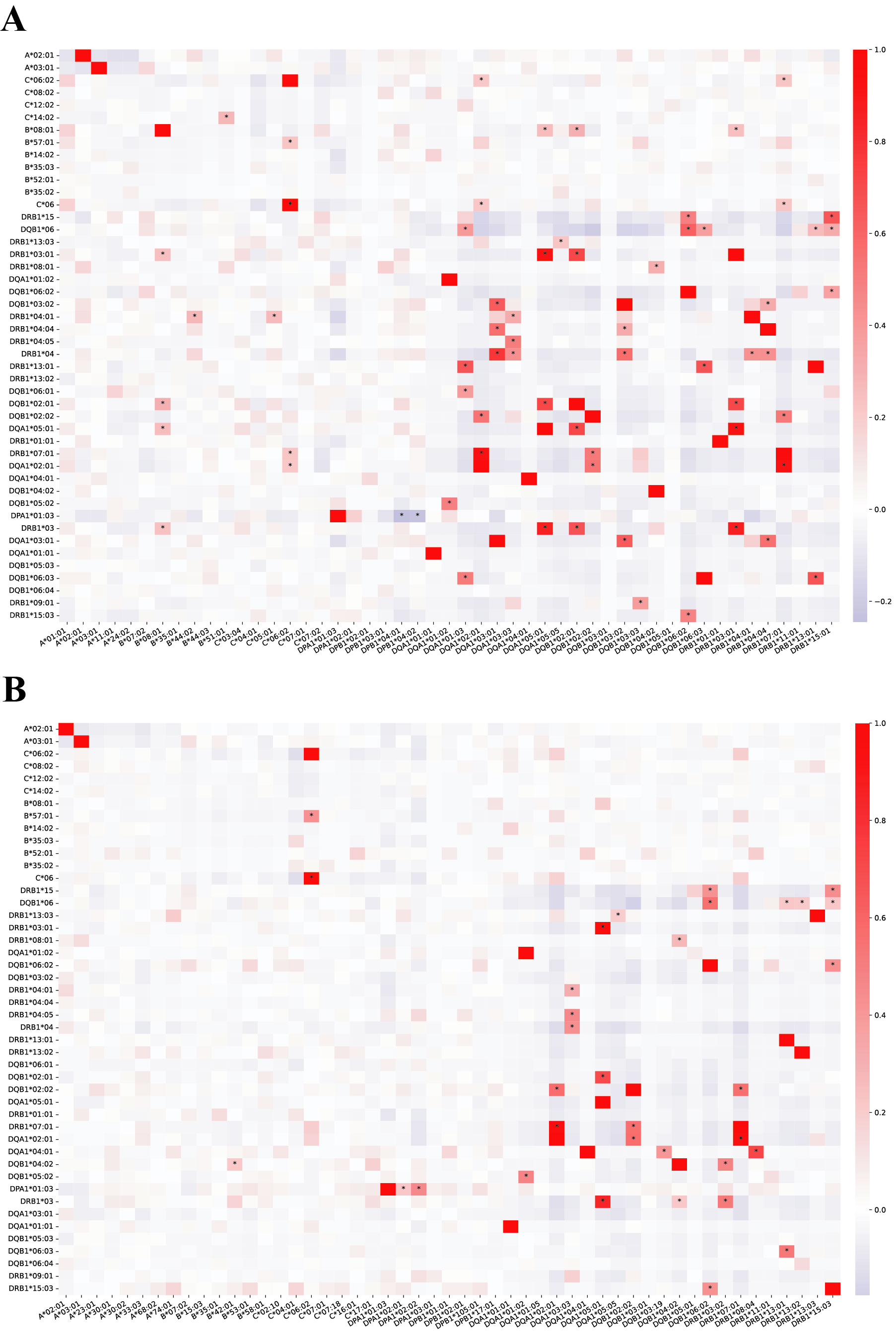

Supplement: qzaf038_Supplementary_Data [file qzaf038_supplementary_data.zip › FigureS10.jpg]

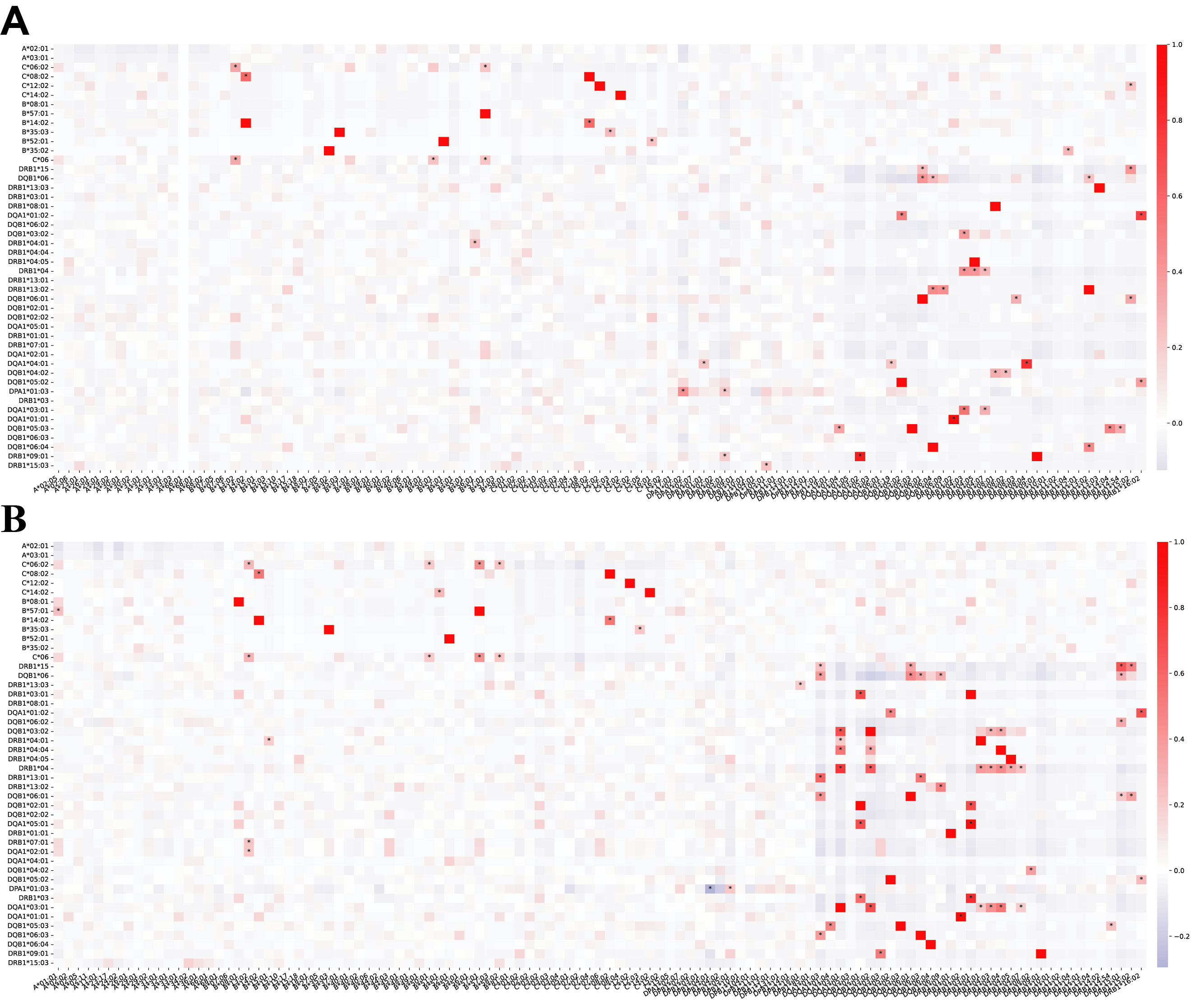

Supplement: qzaf038_Supplementary_Data [file qzaf038_supplementary_data.zip › FigureS11.jpg]

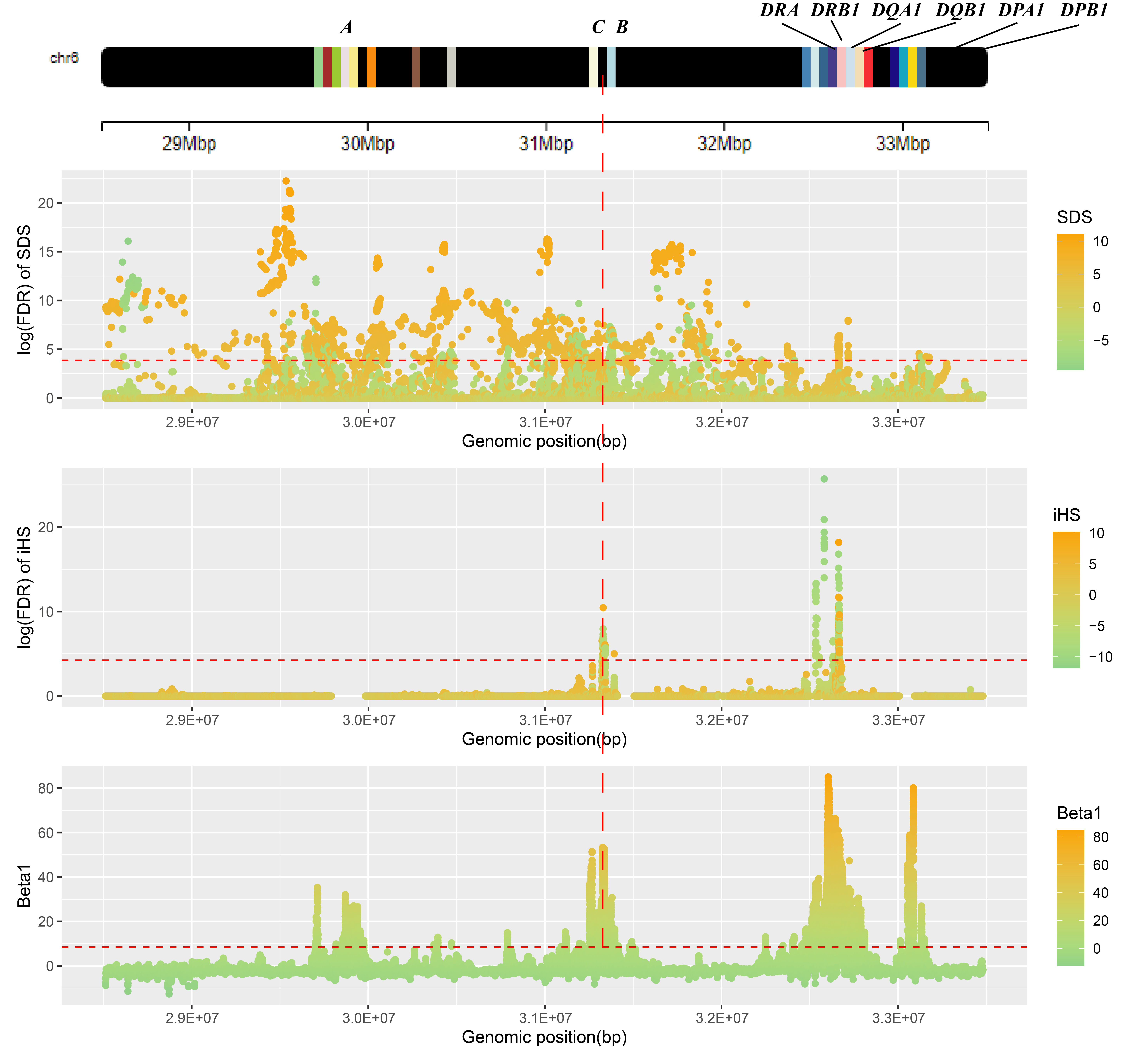

Supplement: qzaf038_Supplementary_Data [file qzaf038_supplementary_data.zip › FigureS12.jpg]

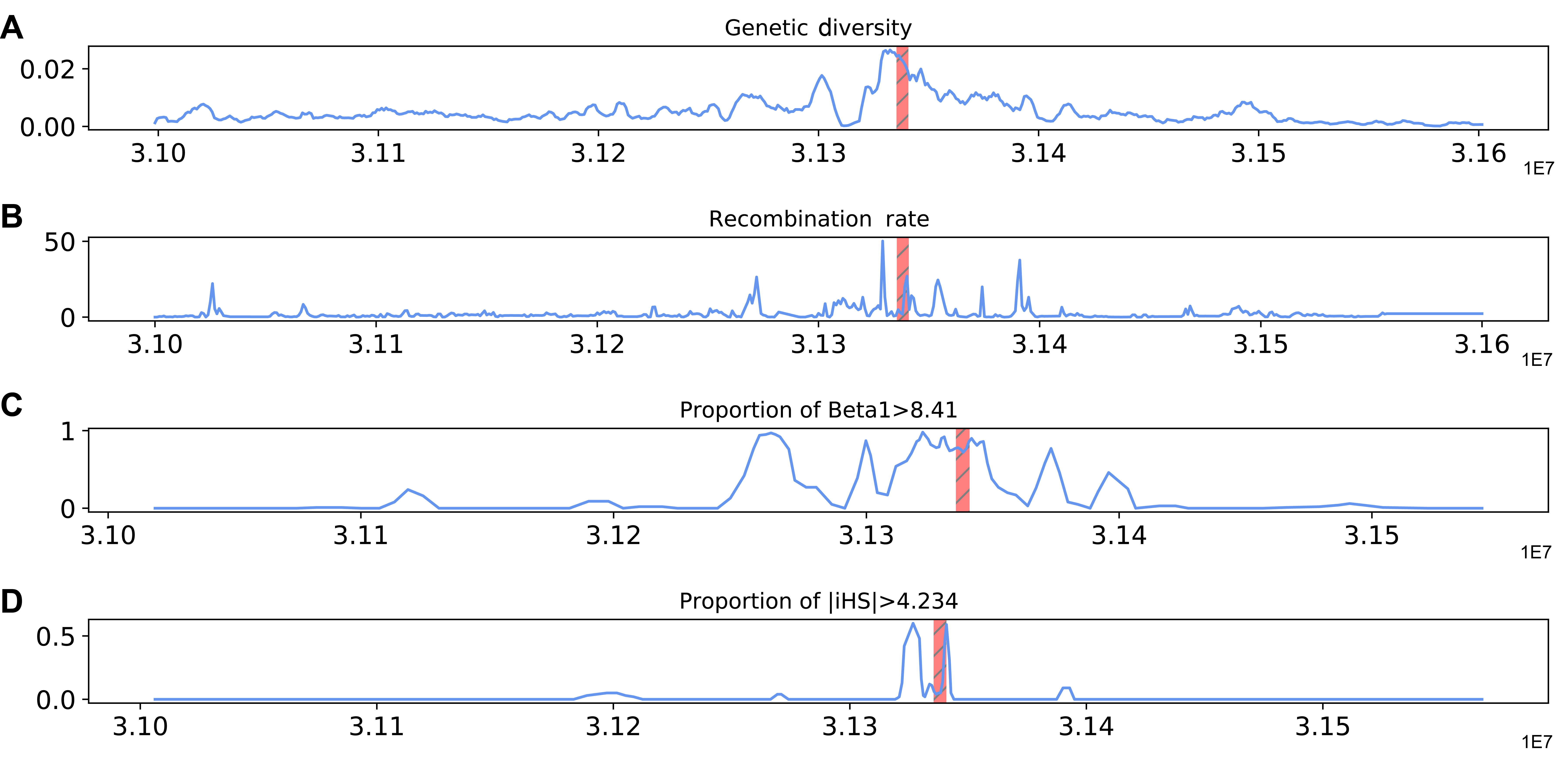

Supplement: qzaf038_Supplementary_Data [file qzaf038_supplementary_data.zip › FigureS13.jpg]

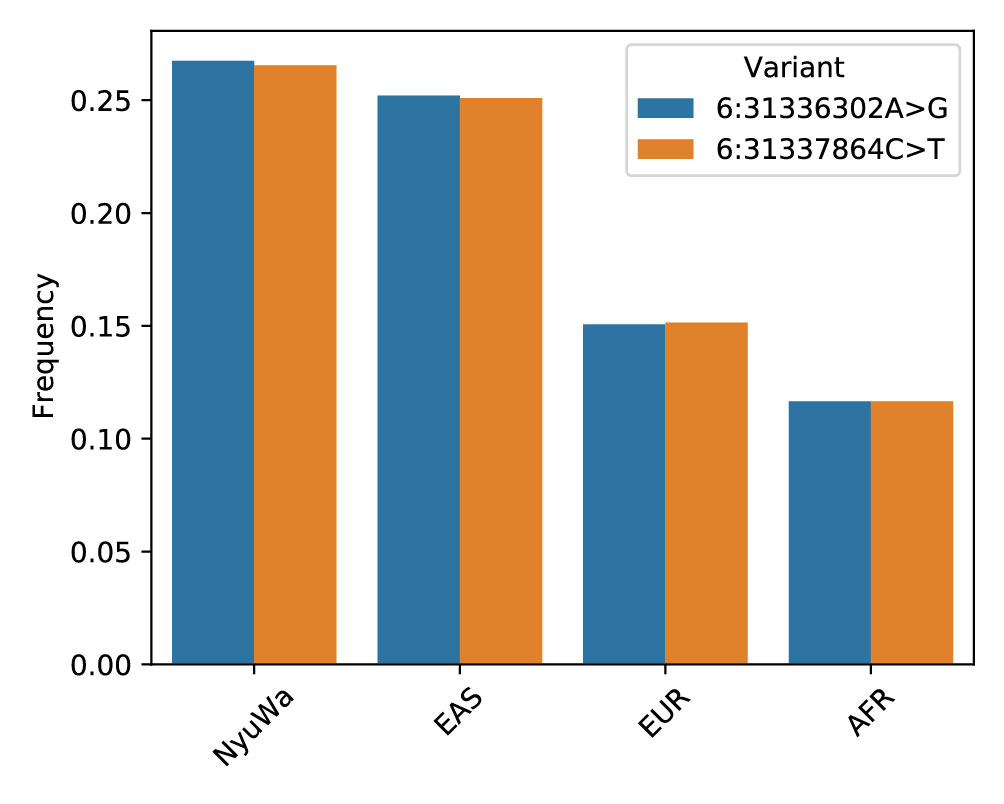

Supplement: qzaf038_Supplementary_Data [file qzaf038_supplementary_data.zip › FigureS14.jpg]

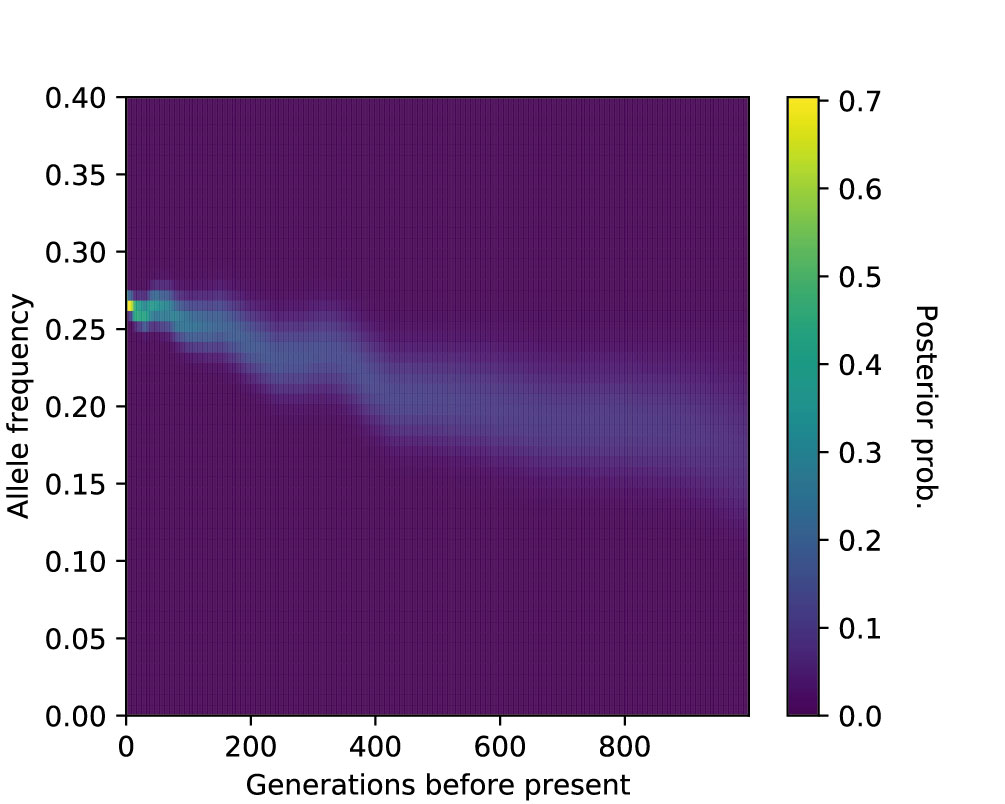

Supplement: qzaf038_Supplementary_Data [file qzaf038_supplementary_data.zip › FigureS15.jpg]

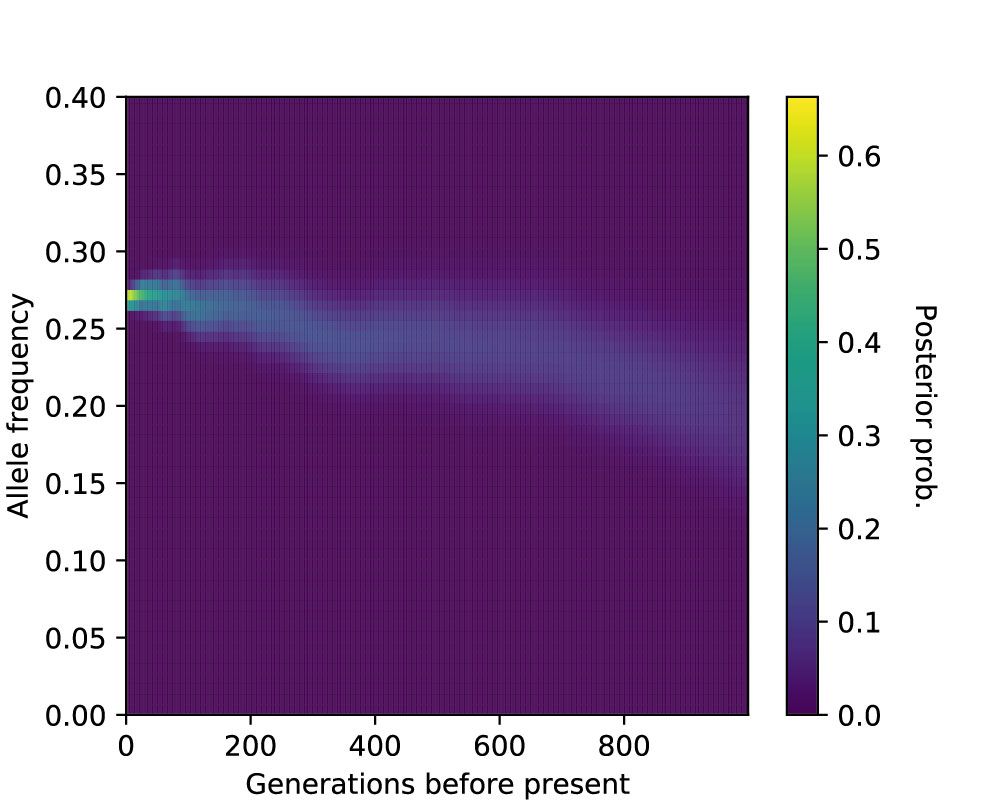

Supplement: qzaf038_Supplementary_Data [file qzaf038_supplementary_data.zip › FigureS16.jpg]

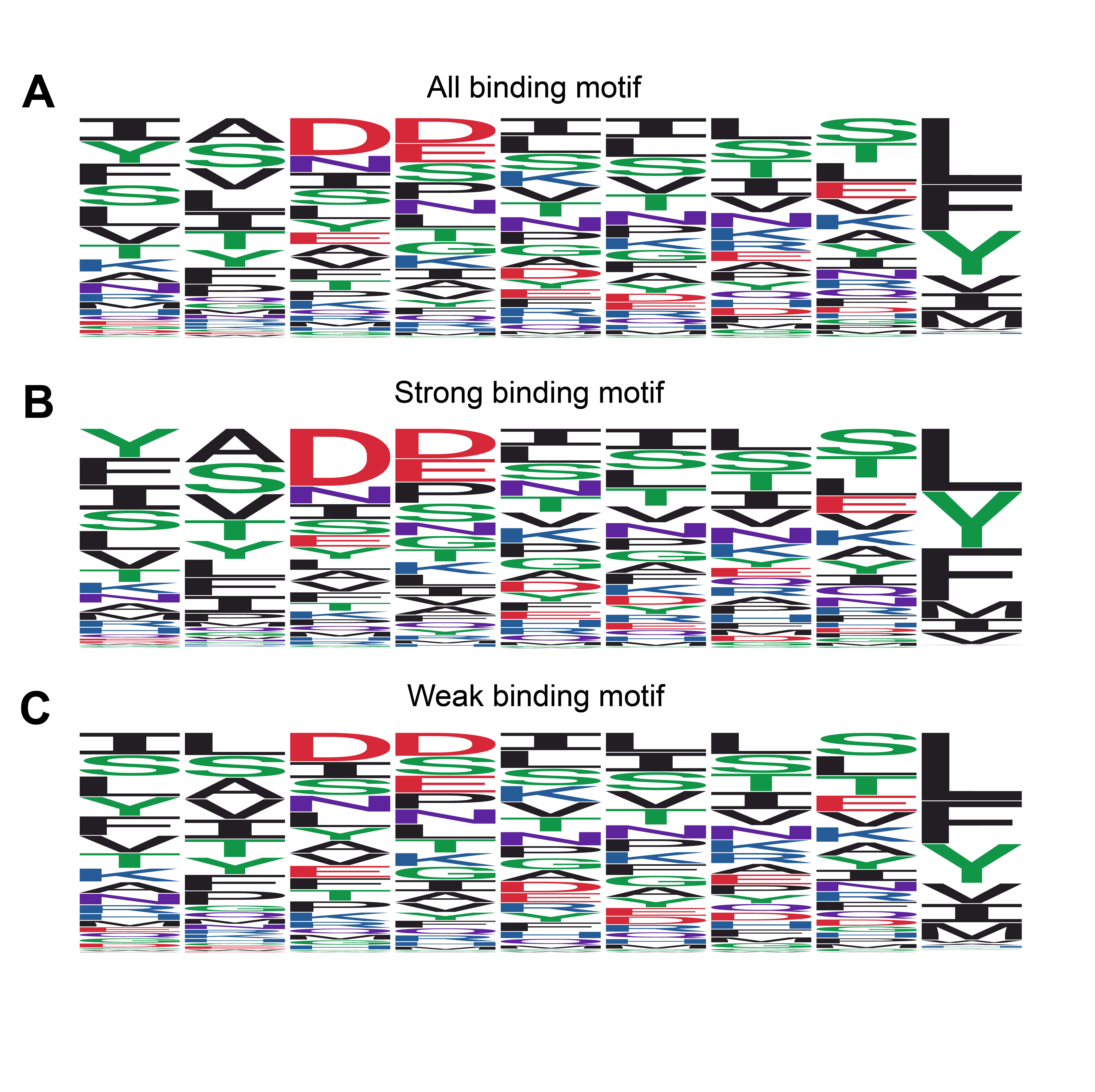

Supplement: qzaf038_Supplementary_Data [file qzaf038_supplementary_data.zip › FigureS17.jpg]

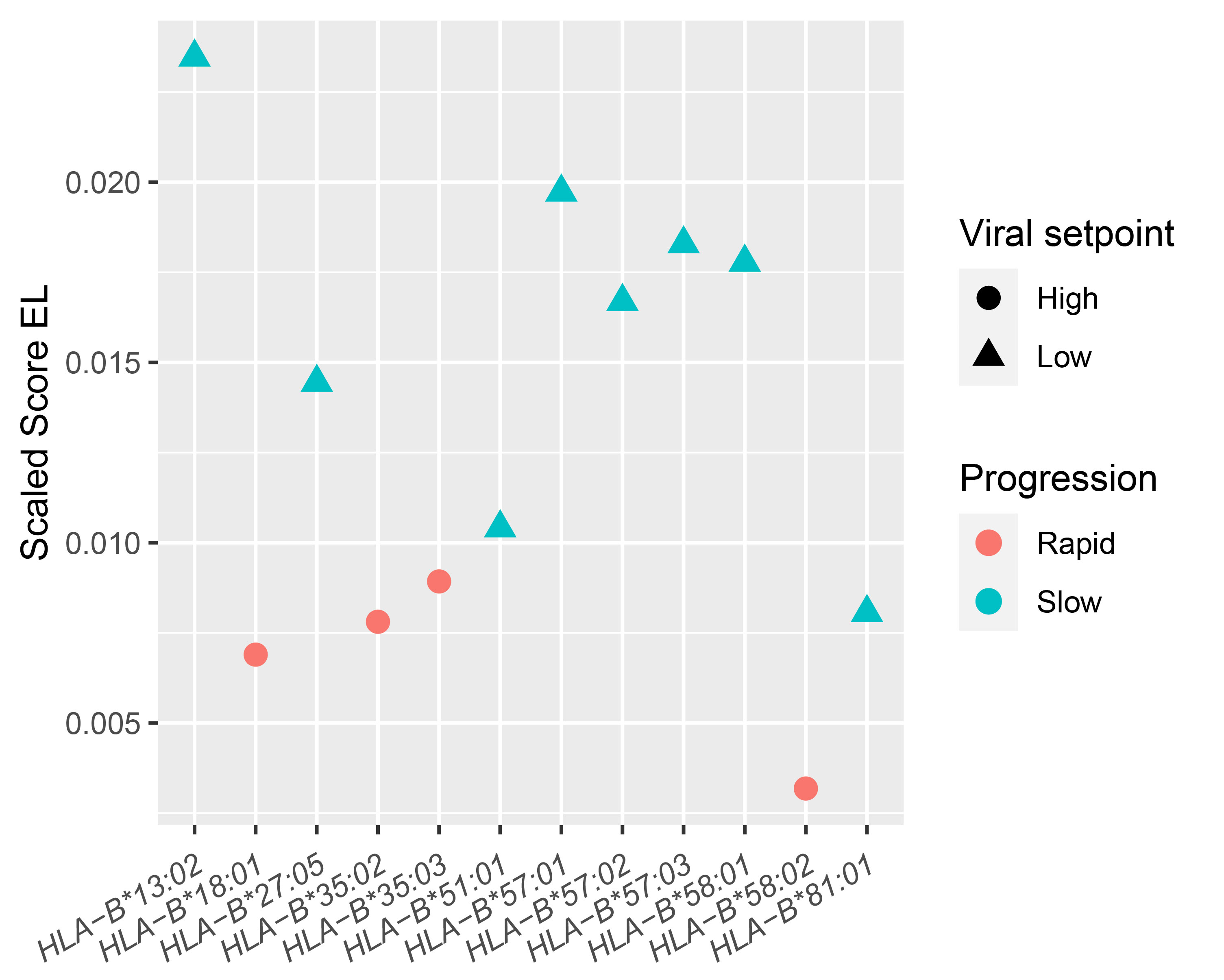

Supplement: qzaf038_Supplementary_Data [file qzaf038_supplementary_data.zip › FigureS2.jpg]

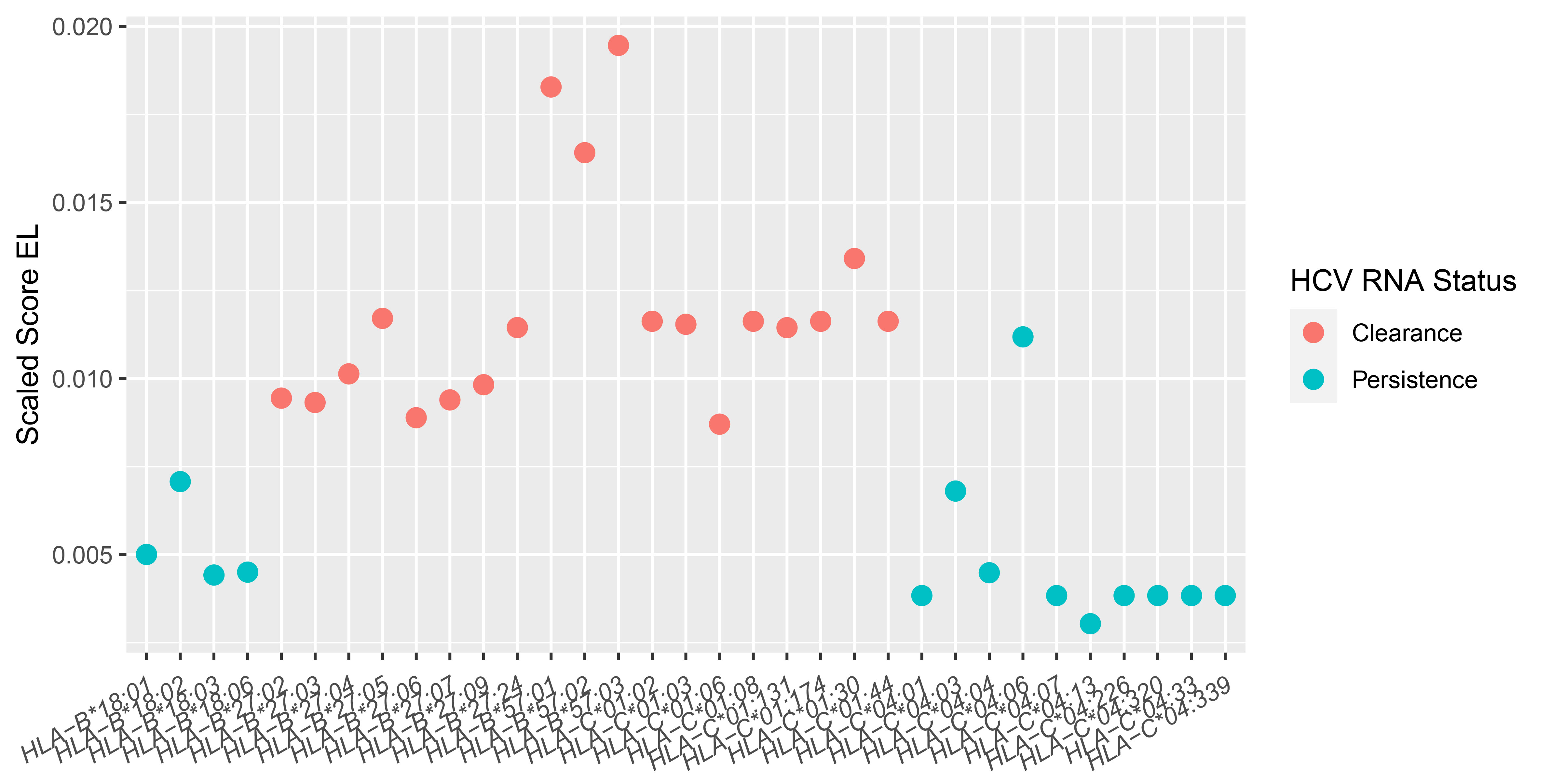

Supplement: qzaf038_Supplementary_Data [file qzaf038_supplementary_data.zip › FigureS3.jpg]

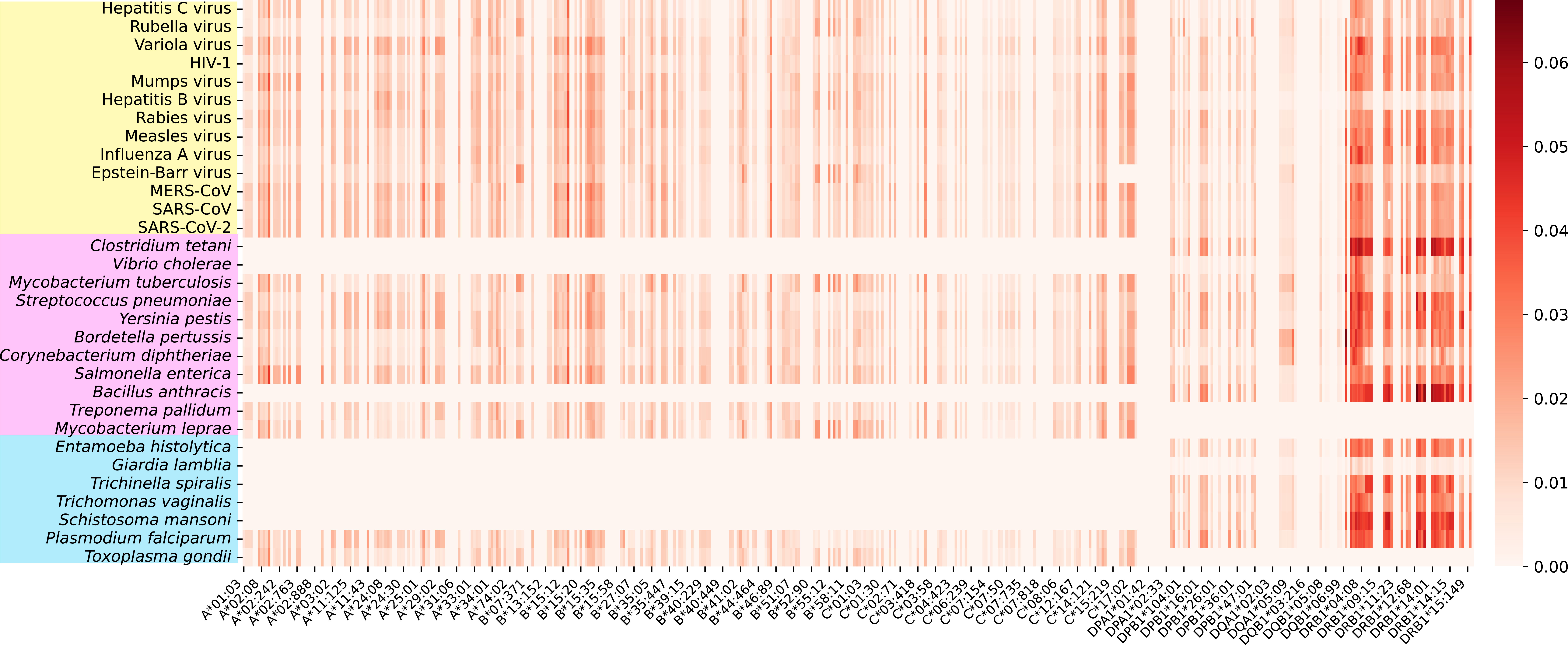

Supplement: qzaf038_Supplementary_Data [file qzaf038_supplementary_data.zip › FigureS4.jpg]

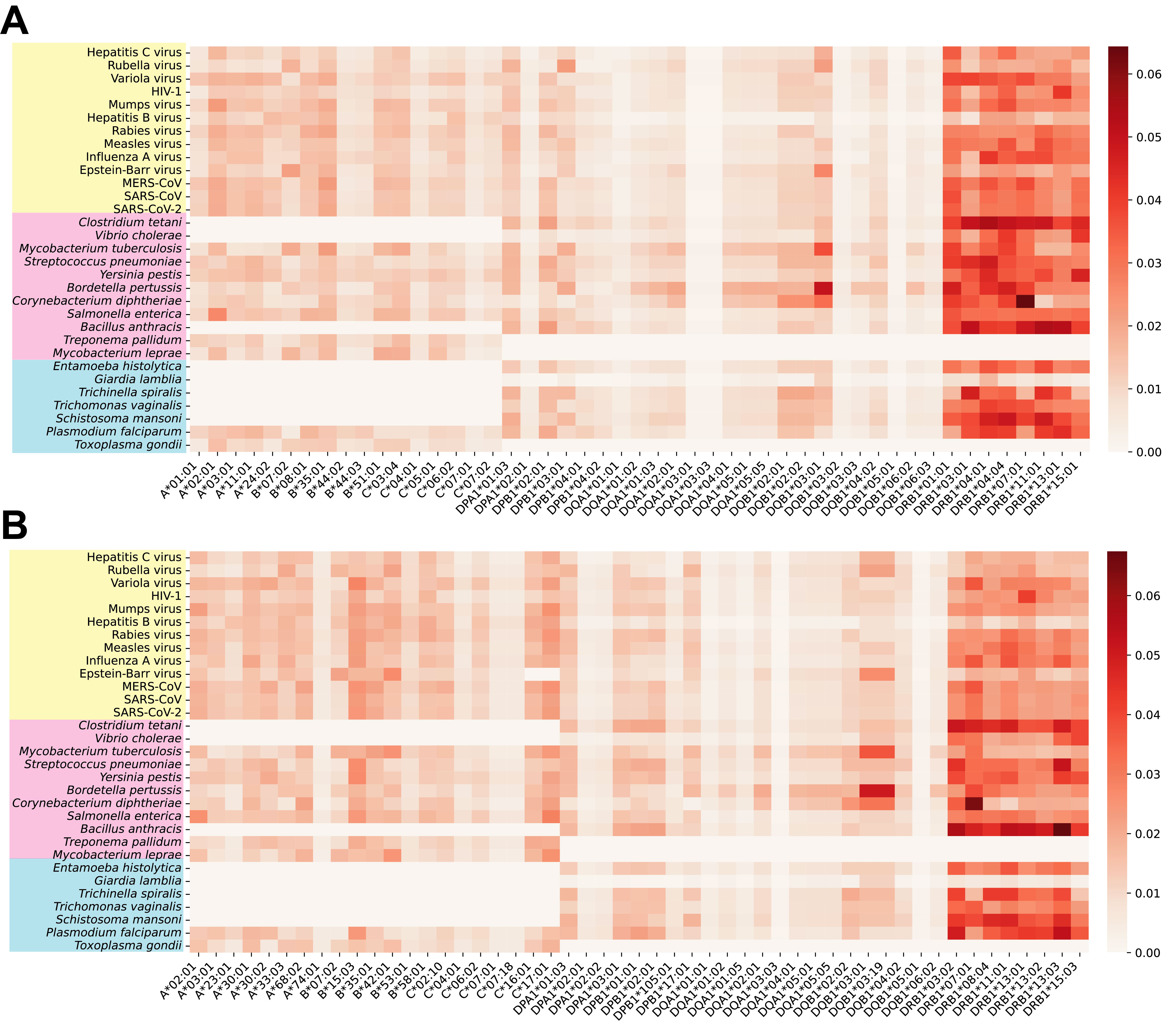

Supplement: qzaf038_Supplementary_Data [file qzaf038_supplementary_data.zip › FigureS5.jpg]

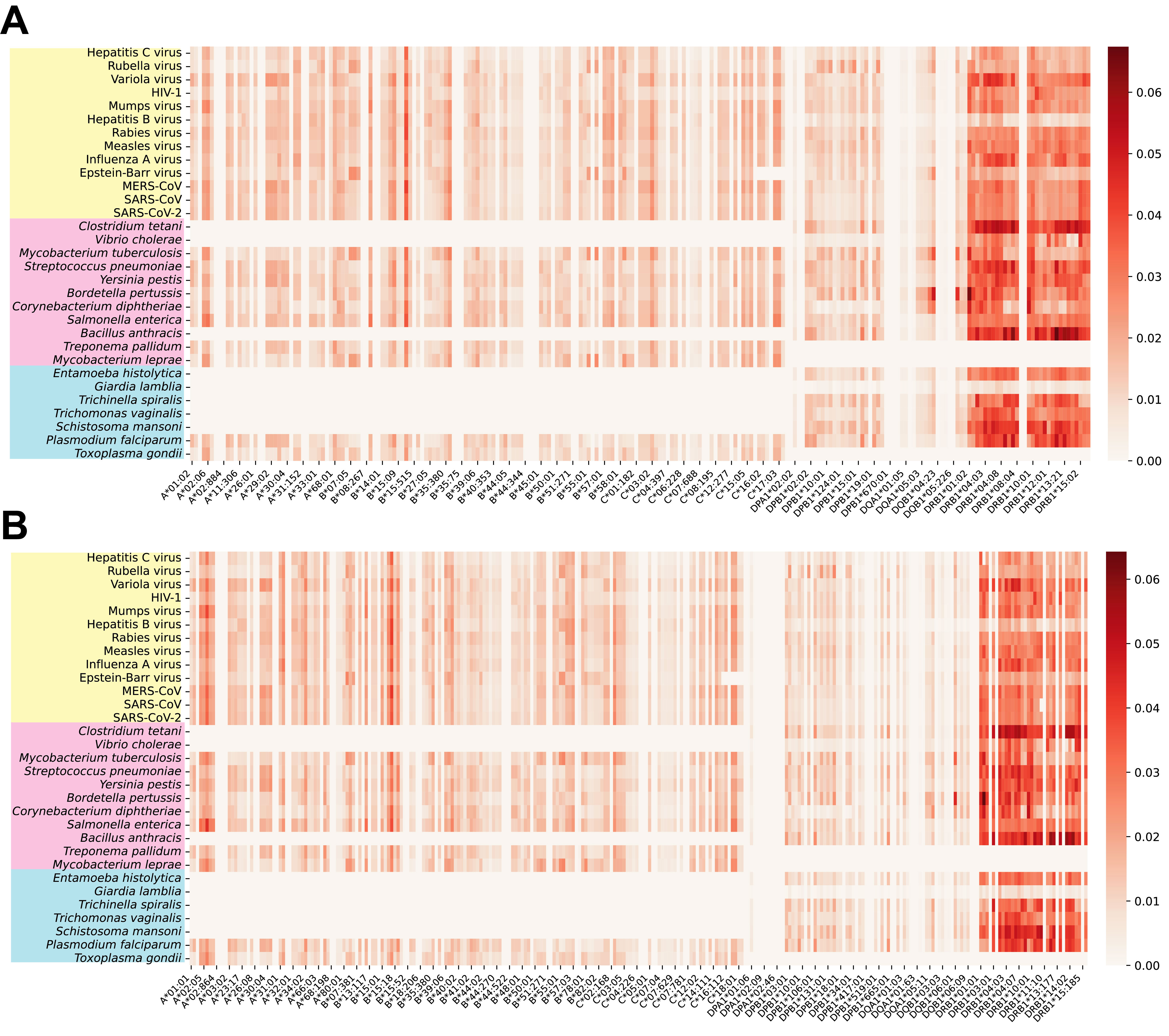

Supplement: qzaf038_Supplementary_Data [file qzaf038_supplementary_data.zip › FigureS6.jpg]

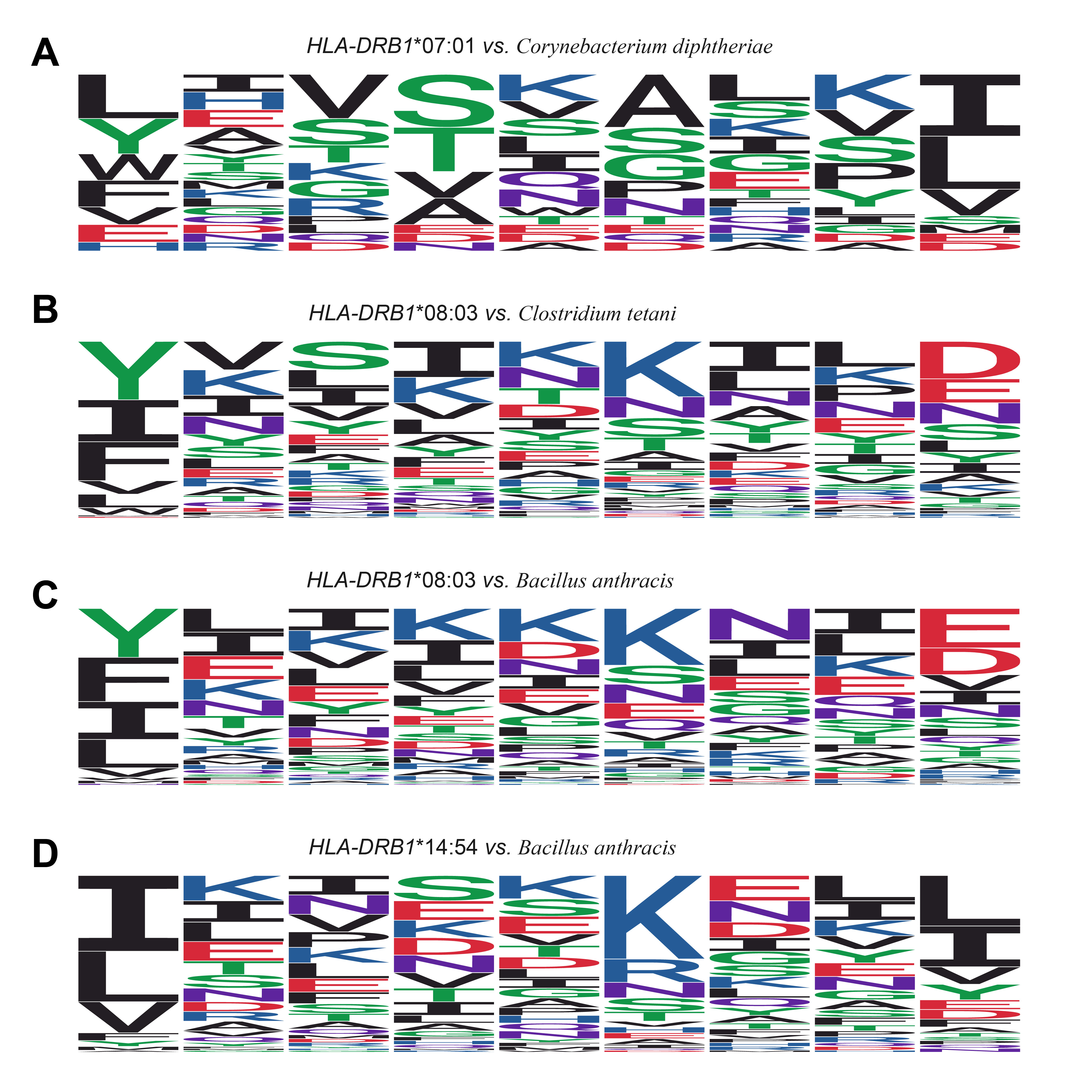

Supplement: qzaf038_Supplementary_Data [file qzaf038_supplementary_data.zip › FigureS7.jpg]

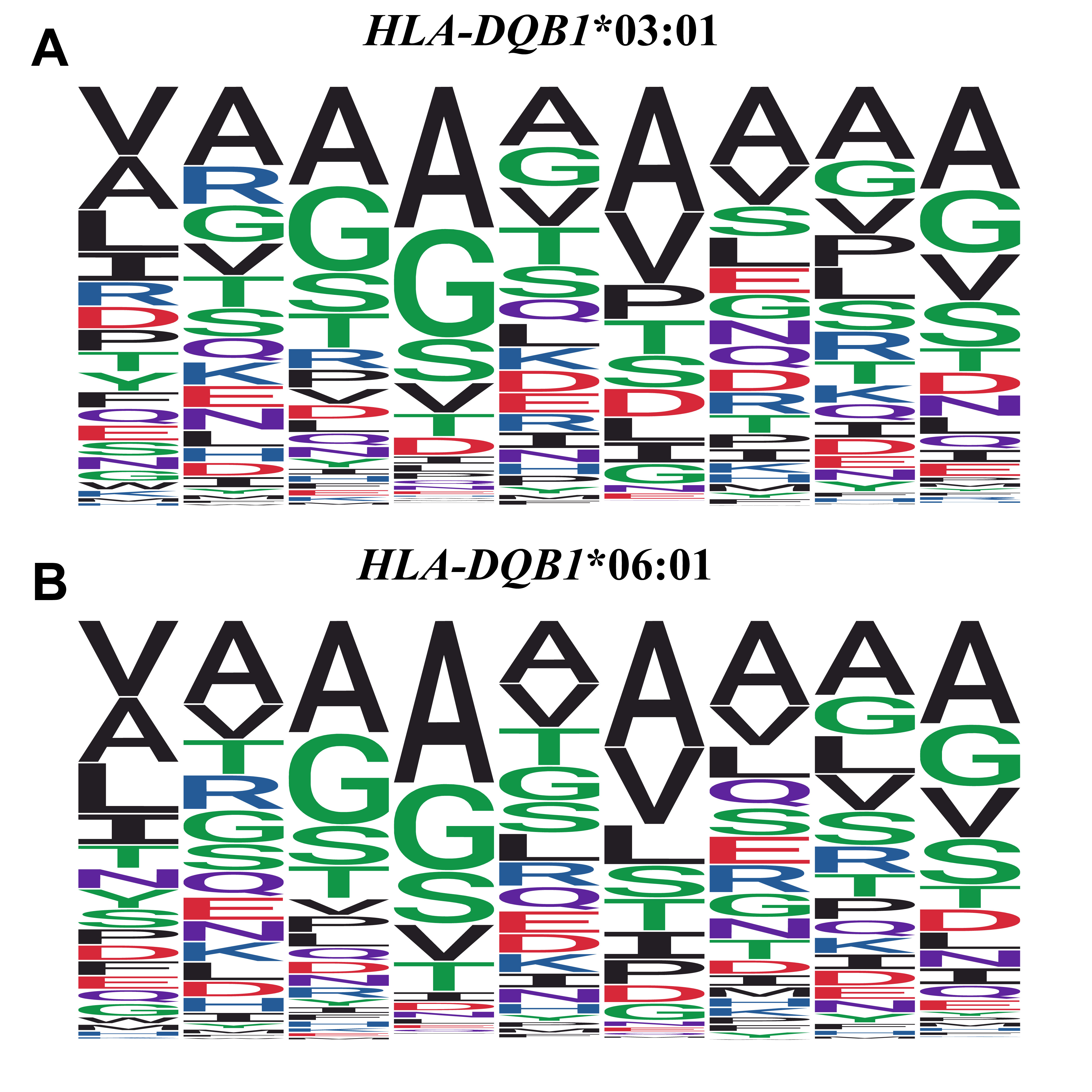

Supplement: qzaf038_Supplementary_Data [file qzaf038_supplementary_data.zip › FigureS8.jpg]
